# HDAC3 inhibition ameliorates ischemia/reperfusion-induced brain injury by regulating the microglial cGAS-STING pathway

**DOI:** 10.7150/thno.47651

**Published:** 2020-07-29

**Authors:** Yajin Liao, Jinbo Cheng, Xiangxi Kong, Shuoshuo Li, Xiaoheng Li, Meijuan Zhang, He Zhang, Tianli Yang, Yuan Dong, Jun Li, Yun Xu, Zengqiang Yuan

**Affiliations:** 1 The Brain Science Center, Beijing Institute of Basic Medical Sciences, No. 27 Taiping Road, Haidian District, Beijing 100850, China.; 2 Center on Translational Neuroscience, College of Life & Environmental Science, Minzu University of China, Beijing 100081, China.; 3 Department of Neurology, Affiliated Drum Tower Hospital of Nanjing University, Medical School, Nanjing 210008, China.; 4 Beijing Institute for Brain Disorders, Capital Medical University, Beijing, 100069, China.; 5 Department of Biochemistry, Medical College, Qingdao University, Qingdao, Shandong, 266071, China.

**Keywords:** Microglia, HDAC3, cGAS, Neuroinflammation, Ischemia/reperfusion

## Abstract

**Rationale:** It is known that neuroinflammation plays a critical and detrimental role in the development of cerebral ischemia/reperfusion (I/R), but the regulation of the cyclic GMP-AMP synthase (cGAS)-mediated innate immune response in I/R-induced neuroinflammation is largely unexplored. This study aimed to investigate the function and regulatory mechanism of cGAS in I/R-induced neuroinflammation and brain injury, and to identify possible strategies for the treatment of ischemic stroke.

**Methods:** To demonstrate that microglial histone deacetylase 3 (HDAC3) regulates the microglial cGAS-stimulator of interferon genes (cGAS-STING) pathway and is involved in I/R-induced neuroinflammation and brain injury, a series of cell biological, molecular, and biochemical approaches were utilized. These approaches include transient middle cerebral artery occlusion (tMCAO), real-time polymerase chain reaction (PCR), RNA sequencing, western blot, co-immunoprecipitation, chromosome-immunoprecipitation, enzyme-linked immunosorbent assay (ELISA), dual-luciferase reporter assay, immunohistochemistry, and confocal imaging.

**Results:** The microglial cGAS- STING pathway was activated by mitochondrial DNA, which promoted the formation of a pro-inflammatory microenvironment. In addition, we revealed that HDAC3 transcriptionally promoted the expression of cGAS and potentiated the activation of the cGAS-STING pathway by regulating the acetylation and nuclear localization of p65 in microglia. Our *in vivo* results indicated that deletion of cGAS or HDAC3 in microglia attenuated I/R-induced neuroinflammation and brain injury.

**Conclusion:** Collectively, we elucidated that the HDAC3-p65-cGAS-STING pathway is involved in the development of I/R-induced neuroinflammation, identifying a new therapeutic avenue for the treatment of ischemic stroke.

## Introduction

Stroke is a devastating cerebrovascular disease which is associated with high morbidity and mortality. Ischemic stroke occurs when the cerebral vasculature is occluded by a thrombus, which results in local brain tissue lacking oxygen and glucose 1, 2. Loss of oxygen and glucose causes neuronal cell death, neuroinflammation, and secondary tissue injury in cerebral hypoperfusion and ischemia/reperfusion (I/R) 3-5. Evidence from an *in vitro* study indicated that a lack of oxygen and glucose could directly induce microglial activation and thereafter activate the type I interferon pathway, which contributes to neuroinflammation 6. In addition, a large number of interferon-stimulated genes (ISGs) are upregulated in brain tissue subjected to I/R 7. The knockout (KO) of either type I interferon-alpha receptor 1 (IFNAR1) or interferon regulatory factor 3 (IRF3) has a neuroprotective effect on transient middle cerebral artery occlusion (tMCAO)-induced brain injury in mice 8, 9. All of these studies indicate that activation of the endogenous type I interferon pathway is detrimental to the outcome of ischemic stroke. However, the mechanism by which the type I interferon pathway is activated remains largely unclear.

The innate immune system prevents infection through a variety of pattern-recognition receptors (PRRs). By recognizing pathogen-associated molecular patterns, PRRs trigger a robust inflammatory response and promote the elimination of pathogens. Among the PRRs, cyclic GMP-AMP (cGAMP) synthase (cGAS) functions as a double-stranded DNA (dsDNA) sensor that survey cytosolic DNA and produces cGAMP. cGAMP then binds to the stimulator of interferon genes (STING) and activates the type I interferon pathway 10-12. Unlike RNA sensors that recognize pathogenic RNAs in a strict structure-dependent manner, cGAS can surveil pathogenic dsDNA irrespective of its sequence and origin 13-15. At the same time, cGAS can be activated by endogenous dsDNA, which may lead to inflammation and autoimmune diseases 16, 17. According to recent studies, cGAS can also be activated by mitochondrial DNA (mtDNA) and micro-nuclear genomic DNA (gDNA) when they are released into the cytoplasm or when cGAS appears in the nucleus 18-20. Upon DNA binding, cGAS becomes activated and initiates germ-free inflammation, promoting the expression of pro-inflammatory factors. For example, cGAS is activated by self-DNA in Aicardi-Goutières syndrome (AGS), and the activation of cGAS leads to severe inflammation in the brain, skin, heart, and muscle 16, 17. Accordingly, inhibition of the cGAS-STING pathway greatly attenuated inflammation and improved symptoms in a mouse model of AGS 16, 21. Therefore, manipulation of the cGAS signaling pathway could be beneficial for cGAS-mediated autoimmune diseases. Previous studies have revealed that post-translational modifications play critical roles in regulating the cGAS-STING pathway, including phosphorylation, glutamylation and ubiquitination 22-24. Competitive inhibition of cGAS has also been discovered in recent years. For example, absent in melanoma 2 competitively inhibits cGAS, which results in caspase 1 and Gasdermin D-mediated cleavage of cGAS and K^+^ efflux 25, 26. However, to date, the mechanisms underlying the transcriptional regulation of cGAS during inflammation have not been elucidated.

Recent evidence has shown that histone deacetylases (HDACs) are involved in I/R-induced brain injury and post-stroke recovery. HDAC2 is activated by oxidative stress post I/R, and inhibition of HDAC2 increases cell viability and neurogenesis post-stroke 27-29. Ischemic preconditioning induces the degradation of HDAC3 in neurons, which enhances their tolerance to I/R 30. Inhibition of HDAC1 and HDAC6 in neurons increases cell viability, whereas inhibition of HDAC4 and HDAC5 decreases cell viability 31, 32. Pan-HDAC inhibitors Trichostatin A (TSA) and vorinostat attenuate ischemic stroke-induced brain injury and promote post-stroke recovery by enhancing neurogenesis 29. Due to the important roles of HDACs in neurological diseases, a large number of pharmaceuticals specifically targeting each individual HDAC are under development. The application of HDAC inhibitors for the treatment of stroke has shown great potential.

Here, we have demonstrated that cGAS is transcriptionally regulated by HDAC and discovered a new HDAC3-p65-cGAS signaling pathway. We first showed that deficiency of cGAS in microglia attenuates neuroinflammation and I/R-induced brain injury. Furthermore, we found that HDAC3 transcriptionally regulates cGAS expression in a p65-dependent manner. Finally, we observed that the deletion or inhibition of HDAC3 in microglia specifically decreased cGAS expression and tissue inflammation, resulting in attenuated pathological injury in a mouse tMCAO model. These results demonstrate that HDAC3-cGAS signaling is essential for ischemic stroke-induced neuroinflammation and tissue injury, identifying a new therapeutic avenue for the treatment of ischemic stroke.

## Materials and Methods

### Animal strains

Generation of cGAS cKO mice: cGAS conditional knockout (cKO) mice were generated at the Model Animal Research Center of Nanjing University using the Clustered Regularly Interspaced Short Palindromic Repeats/Cas9 system. Cas9 mRNA and single-guide RNA were co-injected into zygotes. Then, Cas9 endonuclease cleaved exon 2 (220 base pairs (bp)) of the cGAS gene, creating a double-strand break. These breaks were repaired, and loxP sites were inserted upstream and downstream of exon 2.

HDAC3 cKO mice (Stock No: 024119 | Hdac3^flox^) and CX3CR1^creER-IRES-EYFP^ (also named CX3CR1^creER^) transgenic mice (Stock No: 021160) were purchased from the Jackson Laboratory (Sacramento, CA, USA).

### Tamoxifen-induced knockout of cGAS or HDAC3 in microglia

Tamoxifen (#S1238, Selleckchem, Houston, TX, USA) was dissolved in sunflower oil and 2.5% ethanol to a final concentration of 20 mg/mL and administered to adult mice by gavage (dosage: 4 mg/day, 5 consecutive days). According to a previous study, CX3CR1^creER^ mediated knock-in/knock-out of genes allows for gene manipulation almost exclusively in microglia 33. Therefore, to focus on the function of microglial cGAS and HDAC3 *in vivo*, all associated animal experiments were performed 4 weeks after tamoxifen administration. All mice were maintained in a specific pathogen-free animal facility and handled in accordance with the institute's guidelines for the care and use of laboratory animals.

### Antibodies and reagents

Rabbit anti-cGAS (D3O8O) mAb (#31659, clone: D3O8O, 1:1000 for WB, 1:200 for IP), phospho-IRF-3 (Ser396) (D6O1M) rabbit mAb (#29047, clone: D6O1M, 1:1000), phospho-TBK1/NAK (Ser172) (D52C2) XP® rabbit mAb (#5483, clone: D52C2, 1:1000), acetylated-lysine antibody (#9441S, 1:1000), Rabbit anti-iNOS antibody (#13120, 1:1000) and TBK1/NAK (D1B4) rabbit mAb (#3504, clone: D1B4, 1:1000) were purchased from Cell Signaling Technology (Beverly, MA, USA). Rabbit anti-HDAC3 pAb (H-99) X (#sc-11417 X, 1:1000 for WB, 2 μg of antibody for each chromatin immunoprecipitation (ChIP) sample), rabbit anti-p65 pAb (C-20) (#sc-372, 1:1000 for WB, 2 μg of antibody for each ChIP sample), rabbit anti-IRF3 pAb (FL-425) (#sc-9082, 1:1000), and rabbit anti-histone H2B pAb (FL-126) (#sc-10808, 1:1000) were purchased from Santa Cruz Biotechnology (Dallas, TX, USA). A rabbit anti-p65 (#ab16502, 2 μg of antibody for each ChIP sample) polyclonal antibody and rabbit anti-HDAC1 (#ab7028, 1:1000 for WB, 2 μg of antibody for each ChIP sample), rabbit anti-HDAC2 (#ab12169, 1:2000 for WB, 2 μg of antibody for each ChIP sample), and rabbit anti-HDAC3 (#ab7030, 1:1000 for WB, 2 μg of antibody for each ChIP sample, 1:200 for immunostaining) ChIP-grade polyclonal antibodies were purchased from Abcam (Cambridge, MA, USA). A rabbit anti-STING/TMEM173 polyclonal antibody (#A3575, 1:1000 for WB, 1:200 for IP) was purchased from ABclonal Technology (Wuhan, HB, China). Rabbit anti-acetyl-histone H4 (Lys16) antibody (#17-10101, 1:2000 for WB, 1:1000 for ChIP) was purchased from Merck Millipore (Darmstadt, Hessen, Germany). Rabbit anti-Iba1 polyclonal antibody (#019-19741, 1:1000 for WB, 1:400 for immunostaining) was purchased from FUJIFILM Wako Pure Chemical Corporation (Chuo-ku, Osaka, Japan). Goat anti-Iba1 polyclonal antibody (#NB100-1028, 1:800 for immunostaining) was purchased from Novus Biologicals (Littleton, CO, USA). TRITC-conjugated AffiniPure goat anti-rabbit IgG (#111-025-003, 1:400), Cy5-conjugated donkey anti-mouse IgG (#715-175-150, 1:800), Alexa Fluor 594-conjugated AffiniPure goat anti-mouse IgG (#115-585-003, 1:400), and FITC-conjugated AffiniPure goat anti-rabbit IgG (#111-095-003, 1:400) were purchased from Jackson ImmunoResearch (West Grove, PA, USA). Mouse IL-6 ELISA kit (#431304), PE-labeled anti-CD45 (mouse) antibody (#103105, 1:200), and PerCP/Cy5.5-labeled anti-CD11b (mouse) antibody (#101227, 1:200) were purchased from BioLegend (San Diego, CA, USA).

The HDAC inhibitors TSA (#S1045), MS275 (Entinostat, #S1053), RGFP109 (#S7292), and RGFP966 (#S7229) were purchased from Selleckchem. All inhibitors were prepared as stock solutions in DMSO. NAM (#72340) was purchased from Sigma-Aldrich (St. Louis, MO, USA) and prepared as a stock solution in ddH_2_O. For the *in vivo* study, RGFP966 was dissolved in DMSO to a concentration of 30 mg/mL and diluted in 30% (w/v) hydroxypropyl-β-cyclodextrin (#HK388-5g, Bio Basic Inc., Toronto, ON, Canada) and 100 mM sodium acetate (pH 5.4) to a final concentration of 3 mg/mL before injection. Then, a 30 mg/kg dose of RGFP966 was administered subcutaneously twice per day for 2 consecutive days.

Poly(dA:dT) (#tlrl-patn-1) and cGAMP (#tlrl-nacga23-02) were purchased from InvivoGen (Toulouse, France). Lipofectamine™ 2000 Transfection Reagent (Lipo2000, #11668019) and Lipofectamine™ RNAiMAX Transfection Reagent (RNAi Max, #13778030) were purchased from Life Technologies (Waltham, MA, USA). All primers were synthesized by Life Technologies according to the sequences listed in Supplementary Table S1. All siRNAs were synthesized by Genepharm (Suzhou, JS, China) according to the sequences listed in Supplementary Table S2. Digitonin (#D141), ATP (#FLAAS-1VL), and GTP (#G8877) were purchased from Sigma-Aldrich.

### Cell lines and culture

BV2 microglial cells and HEK293T cells were maintained in Dulbecco's modified Eagle's medium (DMEM) (#11965-092, Life Technologies) supplemented with 10% heat-inactivated fetal bovine serum (FBS, #04-001-1A, Biological Industries, Beit Haemek, Israel) and 1% penicillin-streptomycin solution (#03-031-1B, Biological Industries) at 37 °C in a humidified atmosphere with 5% CO_2_. BV2 cell lines stably expressing short hairpin RNA (shRNA) against HDAC1/2/3and Flag-p65 were constructed by lentivirus-mediated transfection and screened with puromycin. The sequences of the shRNAs against HDAC1, 2, and 3 are listed in Supplementary Table S3.

### Cloning and plasmid construction

To construct the cGAS reporter plasmid, gDNA was extracted from BV2 cells using the EasyPure^®^ Genomic DNA kit (#EE101-01, TransGen Biotech) according to the manufacturer's instructions for mammalian cells. The promoter region of cGAS (from approximately 2000 bp upstream of the ATG codon of cGAS) was amplified by polymerase chain reaction (PCR) with PrimeSTAR GXL DNA polymerase (#R050A, Takara Bio, Dalian, Liaoning, China). The PCR product was purified with a gel extraction kit (#CW2302, CWbiotech, Najing, JS, China) according to the manufacturer's instructions after running the gel. It was then digested with XhoI (#R0146S, New England Biolabs, Ipswich, MA, USA) and HindIII (#R3104T, New England Biolabs) at 37 °C for 2 h and purified with a gel extraction kit. The purified fragment was then ligated with a XhoI and HindIII double-digested pGL3-basic vector and transformed into competent *E. coli* (DH5α) cells. To identify cells containing the plasmid of interest, the cells were cultured in Luria-Bertani medium with ampicillin and subjected to Sanger sequencing. The mutant p65 plasmid was constructed by PCR-mediated site-directed mutagenesis. The pFlag-p65 and mutant plasmids were subcloned from pMyc-p65 to pCDH-EF1-T2A-Puro. All plasmids for transfection were purified using the QIAGEN Plasmid Maxi kit (#12163, QIAGEN, Germantown, MD, USA).

### Dual-luciferase assay

The cGAS reporter plasmid (50 ng/mL) and TK-Renilla plasmid (10 ng/mL) (used as a reference reporter here) were co-transfected into HEK293T cells together with the p65 plasmid. The cells were then treated with HDAC inhibitors or vehicle. The cells were harvested 24 h post-transfection, and Renilla luciferase activity was determined using the Dual-Luciferase Assay System (#E1910, Promega, Madison, WI, USA) and microplate reader (Spark 10M, Tecan, Männedorf, Switzerland).

### Isolation of adult primary microglia

Adult primary microglia were isolated from the cortex and middle brain of adult mice. Briefly, mice were anesthetized and perfused with a 0.9% NaCl buffer. Then, their brains were removed, cut into pieces, and homogenized in ice-cold 2% FBS-containing phosphate-buffered saline PBS in a 2 mL Dounce homogenizer. Then, the homogenate was filtered through a 70 μm strainer and centrifuged at 1000 G at 4 °C for 5 min. Following this, the supernatant was carefully removed, and the pellet was suspended in 2 mL of 35% Percoll solution diluted in PBS (#17-0891-09, GE Healthcare, Boston, MA, USA). Then, 3 mL of 70% Percoll was added to the bottom of a clean 15 mL tube and the suspension was carefully added to the top. Following this, 1 mL 30% Percoll solution and 3mL PBS were added and the solution was intrigued at 2000 G at 4 °C for 20 min. Microglia in the suspension were separated at the interface of the 70% and 30% Percoll solutions, transferred to a clean 15 mL tube, washed with PBS, and stained with a PE-labeled anti-CD45 antibody and PerCP/Cy5.5-labeled anti-CD11b antibody. The microglial population isolated via flow cytometry comprised CD45^low^CD11b^+^ cells. Purified microglia were collected into a 5 mL tube with 2 mL of 2% FBS/RPMI 1640 medium.

### RNA sequencing

Total RNA was extracted from 50000 CD45^low^CD11b^+^ microglia isolated from ischemic brain tissue using a NucleoSpin RNA Plus XS kit (#740990, Macherey-Nagel, Dueren, Germany), and the quality of the extracted RNA was determined using a NanoDrop 2000 spectrophotometer and Qubit 2.0 fluorometer. Adult microglia were isolated from 10 mice from each group. Overall, 100 ng of total RNA was extracted from the microglia of each mouse, and the RNA extracted from mice in the same group was mixed together and divided into 2. cDNA libraries were generated from the mixed RNA using a SMARTer® Universal Low Input RNA Kit for Sequencing (#634938, Takara Bio). RNA sequencing was performed using a NovaSeq6000 and High Output v2 kit (Illumina), and the clean data were analyzed by Anoroad Gene Technology (Beijing, China). The fragments per kilobase million (FPKM) values of each gene from each library were used for Kyoto Encyclopedia of Genes and Genomes (KEGG) analysis and gene-set enrichment analysis (GSEA).

### RNA extraction and real-time quantitative PCR

RNA was extracted from tissue and cells using Trizol^TM^ reagent (#15596026, Life Technologies) according to the manufacturer's instructions. Then, mRNA was converted to cDNA using a One-Step gDNA Removal and cDNA Synthesis kit (AE311-03, TransGen Biotech, Beijing, China) according to the manufacturer's protocol with random primers. Finally, the cDNA was diluted to 5 times the volume of ddH_2_O and used for real-time quantitative PCR (qPCR) with 2× RealStar Green Fast Mixture (with ROX II) (#A304, GenStar, Beijing, China) and STUDIO Q3 (Life Technologies). All real-time qPCR experiments were repeated 3 times.

### Establishment of the mouse transient middle cerebral artery occlusion model

Mice weighing approximately 22 g were anesthetized with an intraperitoneal injection of pentobarbital (2.5%, 10 μL/g body weight). To perform tMCAO, a midline incision was made at the trachea, the soft tissue retracted, and a silicon-coated nylon suture (#602156PK5Re, Doccol, Sharon, MA, USA) was advanced into the MCA for 60 min and the blood flow was monitored by laser Doppler velocimetry (PF5001, PERIMED, Stockholm, Sweden). Mice that underwent sham surgery were used as controls. Rhesus monkey samples were kindly provided by Professor Xunming Ji 34.

### Evaluation of Neurologic deficits

Neurological tests were performed 24 h post-reperfusion using a modified scoring system developed by Longa *et al.* 35. Neurological function was graded on a scale of 0 to 5 as follows: 0 = no deficits; 1 = failure to extend left forepaw fully; 2 = circling to the left; 3 = falling to the left; 4 = no spontaneous walking with a depressed level of consciousness; and 5 = dead.

### Infarct volume measurements

The mice were anesthetized and sacrificed 6 or 24 h post-tMCAO. Blood was collected from the right atrium for serum separation. The brains were harvested after rapid decapitation, cut into 1 mm thick slices, and incubated in a 1.5% 2,3,5-triphenyl tetrazolium chloride solution for 5 min at 37 °C. For each mouse, the infarct volumes of each slice were determined using ImageJ and summed to determine the whole infarct volume.

### Isolation and culture of newborn primary microglia

Primary microglia were isolated from newborn mice as follows: newborn mice were sacrificed, and their cortices were separated in a germ-free environment. Then, the cortices were washed with germ-free PBS, cut into pieces, and digested with 0.25% trypsin (1 mL per brain) at 37 °C for 10 min. An equal volume of 10% FBS/DMEM was added to stop the reaction and the mixture was transferred into a clean 15 mL tube. DNase I was added to the suspension at a final concentration of 100 ng/mL and incubated for 1 min at 37 °C. The suspension was broken down into a single cell suspension via mild pipetting with a 5 mL pipette about 8 times. Then, the sample was centrifuged at 250 G at 4 °C for 5 min and the supernatant was carefully removed. The pellet was suspended in 10% FBS/DMEM containing recombinant mouse nerve growth factor (5 ng/mL, #784004, BioLegend) and recombinant murine fibroblast growth factor-basic (10 ng/mL, #AF-450-33, PeproTech, Rocky Hill, NJ, USA) and was filtered with a 70 μm strainer. Then, the single cell suspension was transferred to a poly L-ornithine-coated T-25 flask (5 mL per flask) and cultured in an incubator (37 °C, 5% CO_2_). After 24 h, the medium was replaced with fresh medium, and this medium exchange was then performed every other day. Eight days later, the microglia were purified by shaking the flask at a speed of 200 rpm for 5 min to separate microglia from astrocytes, and the medium was then transferred into a 15 mL tube and centrifuged at 250 G for 5 min. The supernatant was removed, the pellet was suspended in 1 mL fresh medium, and the number of cells was counted with an automated cell counter (Countess^TM^ II FL, Life Technologies) for seeding the purified microglia to dishes for the functional study. The purity of microglia was confirmed by immunostaining with anti-Iba1 antibody and the percentage of Iba1 positive cells was analyzed by counting.

### Immunostaining and imaging

Slices or fixed cultured cells were permeabilized with 0.5% Triton X-100 and blocked with PBS plus 10% horse serum and 3% bovine serum albumin (BSA). The samples were incubated with primary antibodies (diluted to 1:400 with PBS) overnight at 4 °C. Then, the samples were washed with PBS with Tween 20 (PBST) 4 times and incubated with fluorescently labeled secondary antibodies at 25 °C for 90 min. Finally, the samples were washed once with PBST, the nuclei were stained with 4',6-diamidino-2-phenylindole (#D9542, Sigma-Aldrich), and the samples were washed with PBST a further 3 times. Then, the slices were mounted with ProLong Diamond Antifade Mountant (#P36961, ThermoFisher) and coverslips. Images were captured with a laser confocal microscope (Ti-A1, NIKON, Minato-ku, Tokyo, Japan) and a fluorescent slide scanner (Pannoramic SCAN, 3DHISTECH, Budapest, Hungary). For immunochemistry, samples were pretreated with 0.3% H_2_O_2_ in PBS and the secondary antibody was used according to the user's manual of the VECTASTAIN ABC staining kit (#PK-4001, Vector Laboratories, Burlingame, CA, USA). Images were captured using a slide scanner (SCN400, Leica, Buffalo Grove, IL, USA).

### Oxygen-glucose deprivation/reperfusion (OGD/R)

BV2 microglia cell lines and primary microglia were seeded in a 12-well cell culture plate and cultured in DMEM (high glucose) plus 10% FBS. The medium was removed 12 h after seeding and the cells were washed twice with DMEM (no glucose). Then, the cells were cultured in DMEM (no glucose) at 37 °C in an atmosphere consisting of 95% N_2_ and 5% CO_2_ for 5 h. The medium was replaced with DMEM (high glucose) containing 10% FBS (heat-inactivated) and the cells were cultured for another 2 h to allow reperfusion.

### Immunoprecipitation/real-time qPCR

Newborn brain tissue-derived microglia were subjected to OGD/R for 3 h/2 h. The brain tissue was cut into pieces and homogenized in cold PBS using a Dounce homogenizer. The homogenate was diluted with cold PBS, filtered through a 70 μm strainer, and centrifuged at 1000 G at 4 °C for 5 min. The supernatant was carefully removed and the pellet was suspended in 5 mL PBS. Then, the cells were crosslinked with 1% formaldehyde (#F8775, Sigma-Aldrich) at room temperature for 15 min, and the crosslinking reaction was stopped by adding 0.125 M glycine for 5 min. Following this, the cells were washed and lysed with 5 mL cell lysis buffer (5 mM Pipes (pH 8.0), 85 mM KCl, NP-40 (0.5% v/v), and 1x proteinase cocktail) at 4 °C for 15 min. After centrifugation (4 °C, 12000 G for 15 min), the supernatant was harvested and 20 μL was reserved as input. The rest of the supernatant was used for immunoprecipitation with a rabbit anti-cGAS antibody or IgG from rabbit serum (#I5006, Sigma) overnight at 4 °C. The DNA-protein-IgG complexes were precipitated with protein A magnetic beads (#D10010, Life Technologies) and washed with low salt buffer, high salt buffer, LiCl buffer, and TE buffer. Then, the DNA-protein complex was eluted with elution buffer (1% SDS, 0.1 M NaHCO_3_). The eluents and reserved input were reverse-crosslinked in 100 μL reverse crosslink buffer (0.1 mg/mL RNase and 0.3 M NaCl in ddH_2_O) at 65 °C for 5 h. Then, 2.5 volumes of 100% ethanol were added, and the mixture was precipitated overnight at -20 °C. The DNA pellet and debris were obtained by centrifugation at 16000 G at 4 °C for 15 min, re-suspended in 100 μL nuclease-free water supplemented with 2 μL 0.5 M EDTA, 4 μL 1 M Tris (pH 6.5), and 1 μL 20 mg/mL Proteinase K, and incubated for 2 h at 55 °C. The DNA was then purified using a QIAquick PCR Purification Kit (#28104, QIAGEN) and eluted in 100 μL nuclease-free water. Real-time qPCR analysis was used to measure mtDNA enrichment.

### Cyclic dinucleotide stimulation

cGAMP was diluted in digitonin permeabilization solution (50 mM HEPES [pH 7.0], 100 mM KCl, 3 mM MgCl_2_, 0.1 mM DTT, 85 mM sucrose, 0.2% BSA, 1 mM ATP, 0.1 mM GTP, and 10 μg/mL digitonin) to a final concentration of 100 nM. The medium was aspirated from the cells and replaced with 200 μL of cGAMP solution. The cells were then incubated for 30 min at 37 °C. The medium was again aspirated and fresh BV2 medium (500 μL/well) was added. Four hours after the initial addition of cGAMP solution, the cells were harvested for analysis of the phosphorylation levels of IRF3, STING, and TBK1.

### ChIP assay

The ChIP assay was performed as described in the Abcam X-Chip protocol (https://docs.abcam.com/pdf/protocols/X-ChIP_protocol.pdf). Briefly, the ChIP assay was initiated with 4 dishes of confluent BV2 cells (approximately 2×10^7^ cells/dish). A total of 2 μg of antibody was added to each sample, and the protein-DNA complexes were precipitated using dynamic protein A/G beads (#10001D and #10003D, Life Technologies) pretreated with fatty acid-free BSA (#011-15144, Yisheng, Shanghai, China) and herring sperm DNA (#D1811, Promega).

### Real-time qPCR of cytosolic mtDNA and gDNA

Cytosolic mtDNA was extracted as follows. Briefly, using a Dounce homogenizer, 1×10^7^ cells were homogenized in Tricine-NaOH buffer (100 mM Tricine-NaOH, 0.25 M sucrose, 1 mM EDTA (pH 7.4), and protease inhibitor cocktail) and centrifuged at 700 G for 10 min at 4 °C. The protein concentration and volume of the supernatant were normalized, and the mixture was then centrifuged at 10000 G for 30 min at 4 °C to produce a supernatant corresponding to the cytosolic fraction. DNA was isolated from 200 μL of the cytosolic fraction using a DNeasy Blood & Tissue kit (#69504, QIAGEN). Real-time PCR was performed to measure cytosolic mtDNA and gDNA using 2×RealStar Green Fast Mixture (with ROX II) and established primers for mitochondrial and nuclear genes. The copy numbers of cytosolic mtDNA and gDNA were normalized to that of nuclear DNA by determining the ratio of mtDNA encoding cytochrome c oxidase I to nuclear DNA encoding TERT.

### Separation of cytoplasmic and nuclear proteins

Cells (2×10^6^ cells per sample) were collected, washed with ice-cold PBS, and lysed with 500 μL buffer A (10 mM HEPES-KOH (pH 7.9), 10 mM KCl, 0.1 mM EDTA, 0.1 mM EGTA, 0.125% NP40, and 1x proteinase inhibitor cocktail) for 20 min in a 1.5 mL tube at 4 °C. The cells were agitated for 15 s every 5 min. Then, the cell debris and nuclei were centrifuged at 12000 G at 4 °C for 5 min. The supernatants were carefully transferred to a clean 1.5 mL tube, and the precipitate was washed with 1 mL buffer A. Following this, the precipitate was lysed with 500 μL RIPA (strong) cell lysis buffer. Finally, the proteins in the supernatants (cytoplasmic proteins) and precipitate (nuclear proteins) were analyzed by western blotting.

### Statistical analyses

The error bars displayed throughout the manuscript represent the standard error of the mean and were calculated from 3 technical replicates of each biological sample. For the *in vivo* experiments, error bars were calculated from the average of 3 or more biological replicates. Statistical significance was determined using the unpaired Student's *t*-test for 2 groups under single variance, and one-way ANOVA for multiple groups under single variance or two-way ANOVA for multiple groups under two variances (* indicates *p* < 0.05; ** indicates *p* < 0.01). The data shown are representative of 3 independent experiments for *in vitro* studies and 2 independent experiments for *in vivo* studies.

## Results

### The DNA sensor cGAS is dynamically involved in microglia-mediated neuroinflammation

**Figure 1:**
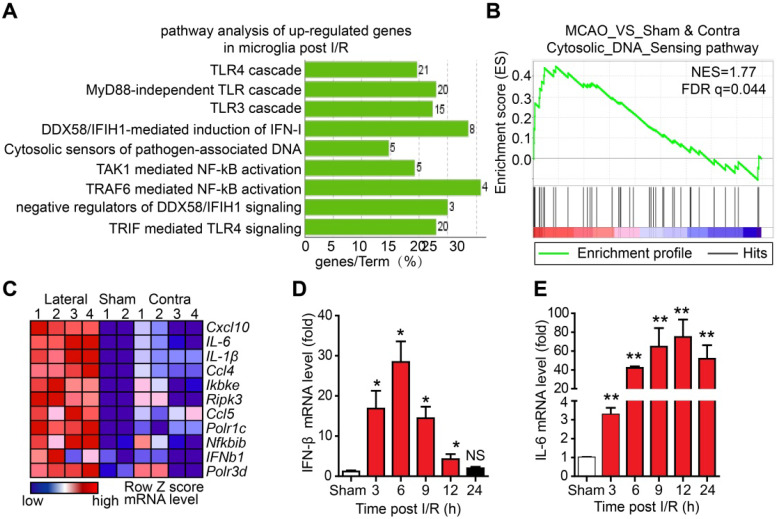
** The cytosolic DNA-sensing pathway is upregulated in microglia after cerebral ischemia/reperfusion (I/R).** (**A**) Kyoto encyclopedia of genes and genomes (KEGG) analysis of the upregulated genes from the RNA sequencing data of adult primary microglia isolated from the brains of mice that underwent tMCAO (10 mice for each sample). (**B**) Gene set enrichment analysis showed that the cytosolic DNA sensing pathway was upregulated in microglia isolated from mice subjected to tMCAO (Sham: the brain underwent sham operation; Contra: the contralateral side of the brain underwent I/R; Lateral: the side of the brain underwent I/R). (**C**) Genes involved in the cytosolic DNA sensing pathway were upregulated in microglia isolated from mice subjected to tMCAO. (**D and E**) Ischemic penumbra cortex was collected from mice that underwent tMCAO at different time points post-reperfusion, and the mRNA levels of IFN-β (D) and IL-6 (E) were quantified by real-time PCR (* indicates* p* < 0.05, ** indicates* p* < 0.01 by ANOVA or Student's *t*-test). Abbreviations: IFN-β, interferon-beta; IL-6, interleukin-6; tMCAO, transient middle cerebral artery occlusion.

Although microglia-mediated inflammation has been shown to be involved in I/R-induced brain injury, the innate immune pathways activated in microglia after I/R and their roles in brain injury are unclear. To elucidate this, we isolated adult microglia from the brains of mice that underwent tMCAO 6 h post-reperfusion. The high-throughput RNA sequencing results of adult microglia isolated from the brains of mice that underwent sham or tMCAO displayed a normal distribution of genes expression (Figure S1A), suggesting that RNA sequencing was well controlled. In addition, the values of FPKM of microglia and monocyte-specific genes were larger than 10, while all the FPKM values of other cell type signature genes were below 1.5 (Figure S1B), indicating a high purity of isolated adult microglia. Further analysis of the upregulated genes by Kyoto encyclopedia of genes and genomes (KEGG) revealed that genes associated with the Toll-like receptor 3/4 cascade, cytosolic sensors of pathogen-associated DNA, and nuclear factor kappa-B (NF-κB) pathway were upregulated (Figure 1A and Figure S1C, D). Interestingly, GSEA suggested that cytosolic DNA-sensing pathway genes were significantly enriched in microglia after I/R (Figure 1B). The leading-edge genes of the DNA-sensing pathway, including *ifnb1*, *il-6*, *cxcl10*,* ccl5, and csf1*, were markedly upregulated in I/R-exposed microglia (Figure 1C and Figure S1E-I). The expression of interferon-beta (IFN-β) and interleukin-6 (IL-6) at different time points post-reperfusion was analyzed. The results showed that the mRNA levels of IFN-β were significantly increased at 3 h post-reperfusion and peaked at 6 h post-reperfusion (Figure 1D). We also observed increased expression of IFN-β in the ischemic penumbra (e.g., the edema region surrounding infarction) from the brain of the Rhesus monkey stroke model (Figure S1J). Similarly, mRNA levels of IL-6 were increased at different time points post-reperfusion (Figure 1E). These results strongly indicate that cytosolic DNA-sensing and type I interferon pathways are activated in microglia after reperfusion.

**Figure 2:**
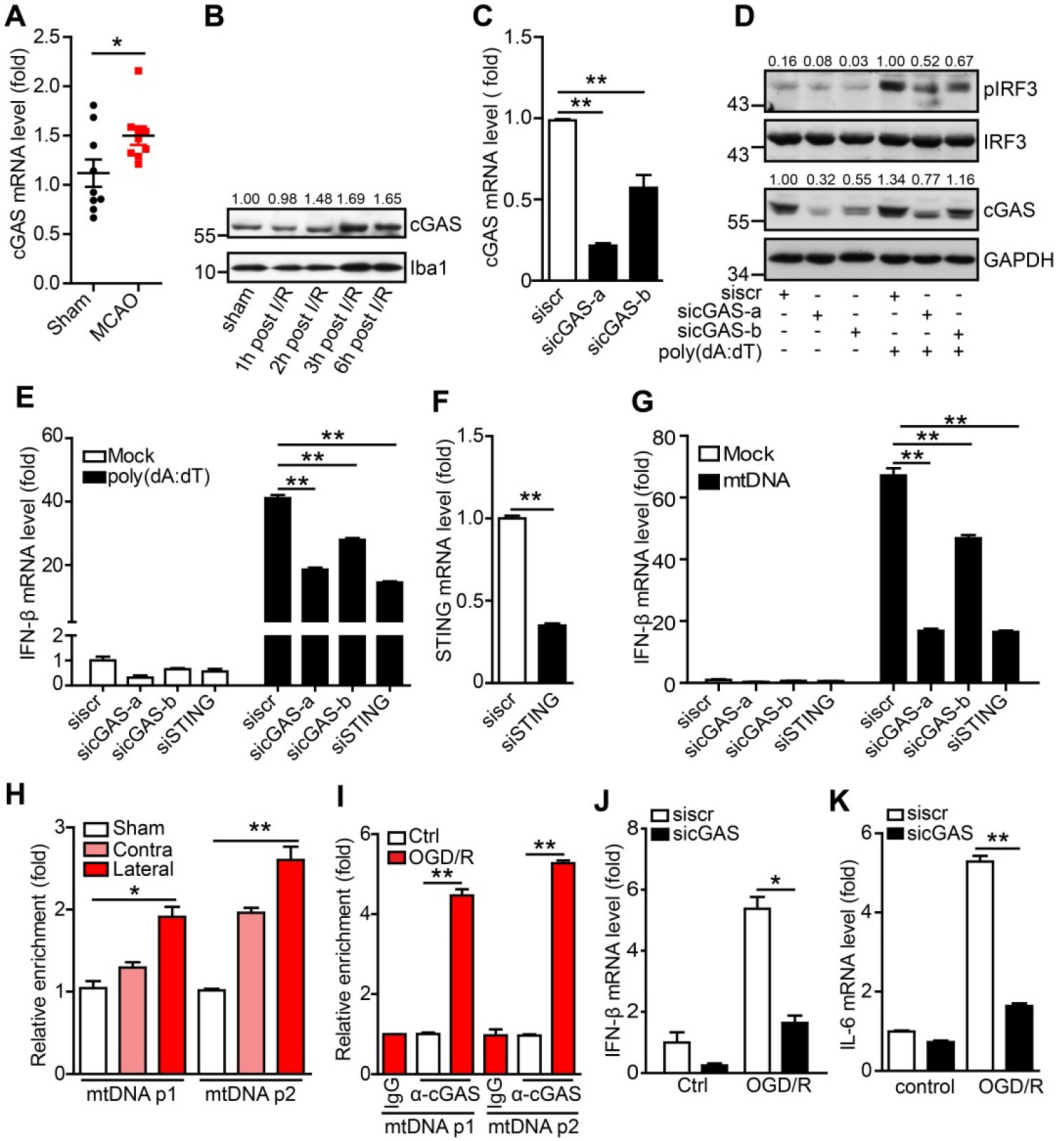
** The cytosolic DNA sensor cGAS is activated after cerebral ischemia/reperfusion.** (**A**) The mRNA level of cGAS in primary microglia isolated from brain tissue 3 h post-tMCAO (9 mice per group). (**B**) The protein level of cGAS in primary microglia isolated from brain tissue at 1, 2, 3 and 6 h post-tMCAO, respectively. The gray values were analyzed by using ImageJ and were normalized to Iba1. (**C**) BV2 cells were transfected with siRNA against cGAS and harvested 48 h post-transfection to quantify the mRNA level of cGAS. (**D**) cGAS-silenced and control BV2 cells were transfected with poly(dA:dT) and harvested 3 h post-poly(dA:dT) treatment to analyze the protein levels of pIRF3, cGAS, and GAPDH. (**E**) cGAS-silenced, STING-silenced, and control BV2 cells were transfected with poly(dA:dT) and harvested 6 h post-poly(dA:dT) treatment to analyze the mRNA level of IFN-β. (**F**) BV2 cells were transfected with siRNA against STING and harvested 48 h post-transfection to quantify the mRNA level of STING. (**G**) cGAS-silenced, STING-silenced, and control BV2 cells were transfected with mtDNA and harvested 6 h post-transfection to analyze the mRNA level of IFN-β. (**H**) The cGAS-mtDNA complex was isolated from brain tissue extracted from mice subjected to tMCAO using an anti-cGAS antibody and quantified by real-time PCR. (**I**) The cGAS-mtDNA complex was isolated from OGD/R-treated primary microglia using an anti-cGAS antibody and quantified by real-time PCR with primer pairs against mtDNA. (**J** and **K**) The mRNA levels of IFN-β (J) and IL-6 (K) in control and cGAS-silenced BV2 cells after OGD/R treatment. (* indicates* p* < 0.05, ** indicates* p* < 0.01 by ANOVA or Student's *t*-test). Abbreviations: cGAS, cyclic GMP-AMP synthase; GAPDH, glyceraldehyde 3-phosphate dehydrogenase; IFN-β, interferon-beta; IL-6, interleukin-6; mtDNA, mitochondrial DNA; OGD/R, oxygen-glucose deprivation/reperfusion; pIRF3, phosphorylated interferon regulatory factor 3; siRNA, small interfering RNA; STING, stimulator of interferon genes; tMCAO, transient middle cerebral artery occlusion.

The DNA sensor cGAS is a cytosolic DNA sensor that can be activated by pathogenic and self-DNA and mediates the activation of the type I interferon and NF-κB pathways. The above results suggest that cGAS may be involved in I/R-induced neuroinflammation and tissue injury. We then examined the expression of cGAS and other cytosolic DNA sensors in adult primary microglia after I/R. The results showed that only cGAS mRNA (Figure 2A), but not the other DNA sensors (Protein LSM14 homolog A and 2'-5'-oligoadenylate synthase 2/3/7), was upregulated 3 h after I/R (Figure S2A). Similarly, we also observed that the transcription level of cGAS increased in the ischemic penumbra from the brain of the Rhesus monkey stroke model (Figure S2B). Consistently, cGAS protein levels were upregulated at the early stage after reperfusion (Figure 2B and Figure S2C).

To check whether cGAS senses and binds to self-DNA after I/R, we first confirmed the role of cGAS in microglia using small interfering RNAs (siRNAs). Both siRNAs (sicGAS-a and sicGAS-b) targeting cGAS significantly inhibited the mRNA and protein levels of cGAS in the BV2 microglial cell line (Figure 2C, D). As expected, cGAS knockdown significantly downregulated poly(dA:dT)-induced IRF3 phosphorylation at S396 (Figure 2D). Moreover, knockdown of cGAS reduced the transcriptional upregulation of IFN-β (Figure 2E), which was similar to the effect of knocking down STING (Figure 2E, F). Next, we analyzed whether microglial cGAS could be activated by self-DNA. Mitochondria were purified from the livers of mice and mtDNA was extracted from purified mitochondria. Then, mtDNA was transfected into microglia using Lipofectamine 2000, and DNase I-pretreated mtDNA was used as a control. Real-time PCR results indicated that the expression of IFN-β in microglia was upregulated by mtDNA transfection. This upregulation was significantly inhibited when cGAS or STING was knocked down (Figure 2G). Moreover, mtDNA-induced IFN-β upregulation was abolished by pretreatment with DNase I (Figure S2D). Together, these results suggest that cGAS is a DNA sensor that mediates the activation of the type I interferon pathway by sensing pathogens and self-DNA in microglia.

To further decipher cGAS activation by mtDNA, the cGAS-DNA complex was purified with an antibody against cGAS from ischemic brain tissue (Figure S2E). Real-time PCR analysis showed that cGAS could bind to mtDNA and that the interaction between mtDNA and cGAS was increased upon I/R (Figure 2H). Furthermore, we observed that the interaction between cGAS and mtDNA was significantly increased in microglial cells exposed to oxygen-glucose deprivation/reperfusion (OGD/R) (Figure 2I). In addition, in OGD/R-treated primary microglia, cGAS knockdown markedly reduced the mRNA levels of IFN-β (Figure 2J) and IL-6 (Figure 2K).

Oxidative stress has also been reported to promote I/R-induced neuroinflammation and brain injury. Here, our results suggest that oxidative stress may enhance cGAS signaling activation by promoting the binding of cGAS to pathogenic mtDNA. In microglial cells, 1-methyl-4-phenylpyridinium (MPP^+^) treatment increased cGAS-mtDNA complex formation (Figure S2F) and oxidative stress and poly(dA:dT) treatment increased the amount of cytosolic mtDNA (Figure S2G). Consistently, MPP^+^ treatment increased the expression of IL-6, and knockdown of cGAS downregulated the MPP^+^-induced expression of IL-6 (Figure S2H). Consistently, deletion of mtDNA with 2,3-dideoxycytidine (Figure S2I) significantly inhibited the activation of the type I interferon pathway (Figure S2J). Taken together, mtDNA released from cells exposed to I/R or oxidative stress promotes the activation of the cGAS-mediated type I interferon pathway in microglia.

### Conditional knockout of cGAS in microglia attenuates I/R-induced neuroinflammation and brain injury

**Figure 3:**
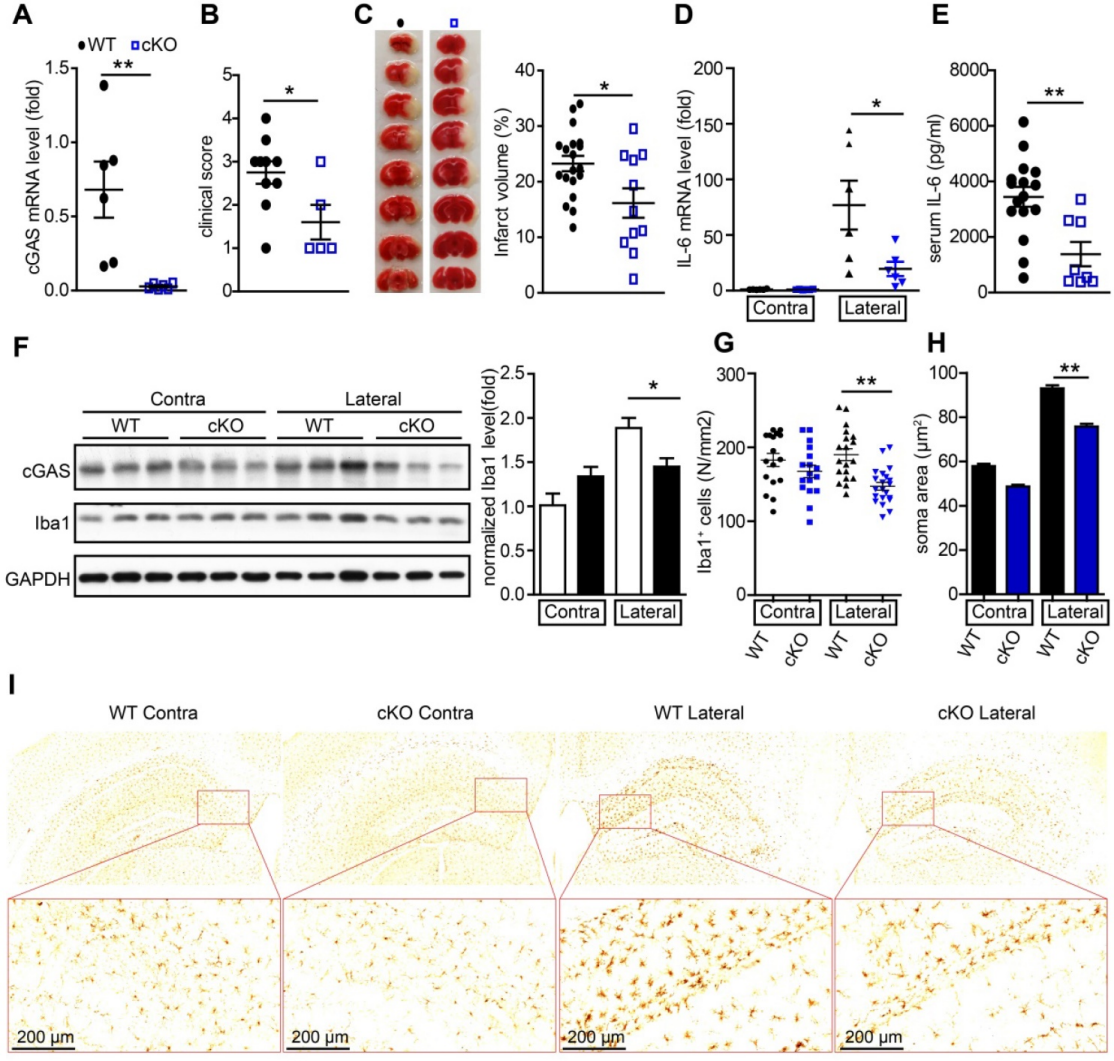
** Conditional knockout of cGAS in microglia attenuates ischemic/reperfusion-induced brain injury.** (**A**) Adult primary microglia were isolated from WT (n = 6) and cGAS cKO (n = 6) mice 2 weeks after tamoxifen administration, and the mRNA level of cGAS was quantified by real-time PCR. (**B**) The neurological deficits of WT (n = 10) and cGAS cKO (n = 5) mice were assessed 24 h post-tMCAO. (**C**) Brain tissue isolated from WT (n = 19) and cGAS cKO (n = 11) mice 24 h post-reperfusion was stained with triphenyl tetrazolium chloride, and infarct volume was determined using ImageJ. (**D**) The mRNA level of IL-6 in the ischemic and contralateral cortices of WT (n = 6) and cGAS cKO (n = 6) mice 6 h post-tMCAO. (**E**) The protein level of IL-6 in serum from WT (n = 16) and cGAS cKO (n = 8) mice 6 h post-tMCAO. (**F**) The protein levels of cGAS and Iba1 in the ischemic penumbra brain tissue were detected by western blot and analyzed with the ImageJ. software (**G** to **I**) The number (**G** and **I**) and soma area (**H** and **I**) of Iba1^+^ cells in the contra and ischemic penumbra of cGAS cKO and WT mice subjected to tMCAO 6 h post-reperfusion. (* indicates* p* < 0.05, ** indicates* p* < 0.01 by ANOVA or Student's *t*-test). Abbreviations: cGAS, cyclic GMP-AMP synthase; cKO, conditional knockout; Iba1, ionized calcium-binding adapter molecule 1; IL-6, interleukin-6; tMCAO, transient middle cerebral artery occlusion; WT, wild-type.

To further study the *de novo* role of cGAS in I/R-induced brain injury, we generated cGAS^f/f^:CX3CR1^creER/+^ mice by crossing cGAS^f/f^ mice with CX3CR1^creER/+^ mice. In these mice, tamoxifen administration induced the cKO of cGAS in microglia (Figure S3A). To confirm the knockout efficiency, we purified primary microglia from cGAS^f/f^:CX3CR1^creER/+^ mice 2 weeks after tamoxifen treatment to analyze the mRNA and protein levels of cGAS. The results showed that the mRNA levels (Figure 3A) and protein levels (Figure S3B) of cGAS in primary microglia was significantly reduced. Four weeks after administration of tamoxifen to restore the expression of cGAS in macrophages, we subjected wild-type (WT) and cGAS cKO mice to I/R injury and found that microglial deletion of cGAS significantly reduced neurological deficit score (Figure 3B) and infarct volume 24 h post-I/R (Figure 3C). cGAS cKO also dramatically reduced the mRNA levels of IL-6 and IFN-β in the ischemic penumbra of mice subjected to tMCAO (Figure 3D and Figure S3C). Consistently, the mRNA level of IFN-β was upregulated in the primary microglia isolated from the brain of WT mice that underwent tMCAO, which was reduced by knockout of cGAS (Figure S3D). Moreover, the protein levels of IL-6 in serum from mice exposed to tMCAO decreased when cGAS was deleted in microglia (Figure 3E). Consistently, reduced microglial activation was noted in the ischemic penumbra of mice with microglial cGAS-deficiency after I/R (Figure 3F-L and Figure S3E). Together, these results indicate that the cGAS-STING pathway is involved in I/R-induced neuroinflammation and brain injury, and deletion of cGAS in microglia can attenuate I/R-induced brain injury.

### HDAC3 is important for the transcriptional regulation of cGAS in microglia

**Figure 4:**
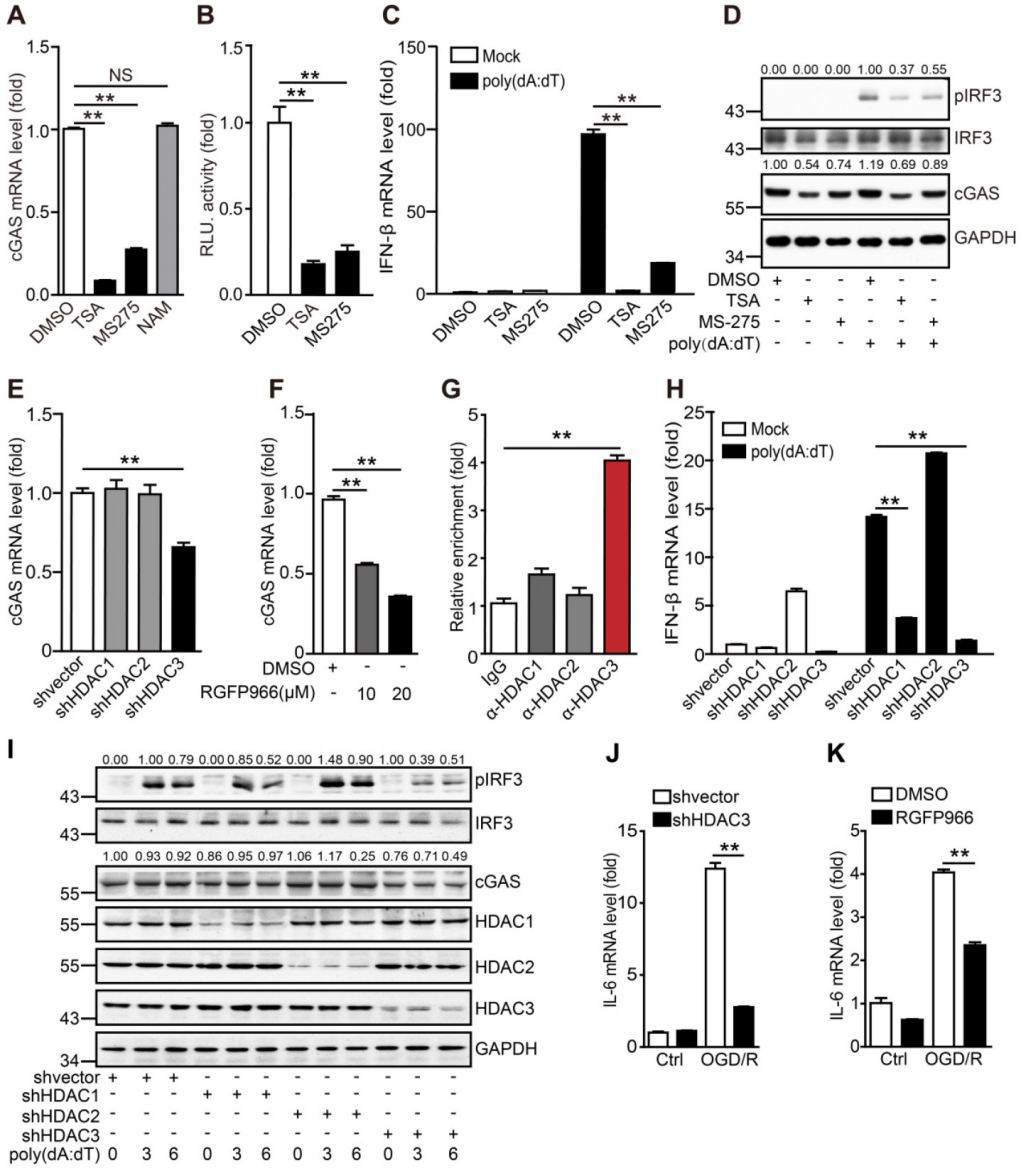
** HDAC3 promotes the transcription of cGAS in microglia** (**A**) The mRNA levels of cGAS in TSA-, MS275-, and NAM-treated BV2 cells were quantified by real-time PCR. (**B**) The cGAS reporter plasmid and TK-Renilla reference reporter plasmid were co-transfected into HEK293T cells and the cells were treated with TSA, MS275, or vehicle for 24 h. (**C** and **D**) BV2 cells were pretreated with TSA, MS275, or vehicle for 12 h and poly(dA:dT) (0.5 μg/mL) was then transfected into the cells using Lipofectamine 2000. For real-time PCR, cells were harvested 6 h post-transfection (C). For western blotting, cells were harvested 3 h post-transfection (D). (**E**) BV2 cells stably expressing shRNA against HDAC1, HDAC2, and HDAC3 were collected and the mRNA levels of cGAS were analyzed by real-time PCR. (**F**) BV2 cells were treated with RGFP966 and the mRNA level of cGAS was determined by real-time PCR. (**G**) The enrichment of HDAC1, HDAC2, and HDAC3 in the promoter region of cGAS in BV2 cells was analyzed by chromatin immunoprecipitation followed by real-time PCR with antibodies against each HDAC. (**H** and **I**) Control and HDAC1-silenced, HDAC2-silenced, HDAC3-silenced, BV2 cell lines were transfected with poly(dA:dT) and harvested 0, 3, and 6 h post-transfection for real-time PCR (H) and western blot (I). (**J**) The mRNA level of IL-6 in control and HDAC3-silenced BV2 cells after OGD/R treatment. (**K**) The mRNA level of IL-6 in vehicle- and RGFP966-pretreated cells after OGD/R treatment. (* indicates* p* < 0.05, ** indicates* p* < 0.01 by ANOVA or Student's* t*-test). Abbreviations: NAM, nicotinamide; cGAS, cyclic GMP-AMP synthase; HDAC, histone deacetylase; IL-6, interleukin-6; OGD/R, oxygen-glucose deprivation/reperfusion; shRNA, short hairpin RNA; TSA, trichostatin A.

It has been reported that HDAC9 promotes the activation of the type I interferon pathway by deacetylating TANK-binding kinase 1 (TBK1). Microglia are the resident innate immune cells of the central nervous system, and it remains unclear whether HDACs regulate the cGAS-STING pathway in microglia under physiological and pathological conditions. Here, we found that the commonly used HDAC inhibitors TSA and MS275, but not NAM, inhibited cGAS transcription in microglia (Figure 4A). We then cloned the cGAS promoter and constructed a dual-luciferase reporter system to detect the transcriptional activation of cGAS. We confirmed that TSA and MS-275 could directly suppress cGAS transcription (Figure 4B). As expected, the mRNA levels of IFN-β (Figure 4C) and phosphorylation of IRF3 (Figure 4D) induced by poly(dA:dT) were also inhibited. Based on the shared targets of TSA and MS275, we hypothesized that HDAC1, HDAC2, and HDAC3 are most likely to regulate cGAS transcription. To test this hypothesis, HDAC1, HDAC2, and HDAC3 were individually knocked down in BV2 cells (Figure S4A). Notably, only the HDAC3 knockdown significantly reduced cGAS mRNA levels (Figure 4E). Consistently, mRNA levels of cGAS were reduced by RGFP966 and RGFP109, 2 commercial HDAC3 inhibitors (Figure 4F and Figure S4B). In addition, HDAC3 was enriched in the promoter region of cGAS (Figure 4C, G). Importantly, HDAC3 knockdown attenuated poly(dA:dT)-induced upregulation of IFN-β (mRNA) (Figure 4H). Moreover, IRF3 phosphorylation levels and cGAS protein levels were significantly decreased when HDAC3 was silenced (Figure 4I). Consistently, poly(dA:dT)-induced upregulation of IFN-β (mRNA) was markedly inhibited by the HDAC3 inhibitor RGFP966 (Figure S4D) and RGFP109 (Figure S4E). RGFP109 also significantly reduced poly(dA:dT)-induced upregulation of IRF3 phosphorylation and cGAS protein levels (Figure S4F). Together, we showed that HDAC3 can transcriptionally regulate the expression of cGAS.

In addition to poly(dA:dT), OGD/R also significantly induced immunoactivation, which was blocked by HDAC3 knockdown (Figure 4J and Figure S4G, H) and RGFP966 (Figure 4K and Figure S4I). In summary, these results indicate that HDAC3 transcriptionally regulates cGAS expression and participates in I/R-induced neuroinflammation through the cGAS-STING pathway.

Interestingly, in addition to the transcriptional regulation of cGAS expression, we found that HDAC3 also acts downstream of cGAS, since HDAC3 knockdown and inhibition significantly reduced the phosphorylation of STING and IRF3 by cGAMP, the effector of cGAS activation (Figure S5A-C). The co-immunoprecipitation (Co-IP) assay showed that HDAC3 inhibition significantly decreased the cGAMP-stimulated phosphorylation of STING and IRF3 within the STING-TBK1-IRF3 complex in microglia (Figure S5D). These results suggest that HDAC3 may also act downstream of the cGAS-STING pathway (Figure S5E). However, the exact mechanism underlying the regulation of HDAC3 in the STING-TBK1-IRF3 complex requires further study.

### HDAC3 promotes the transcription of cGAS by deacetylating p65 in microglia

**Figure 5:**
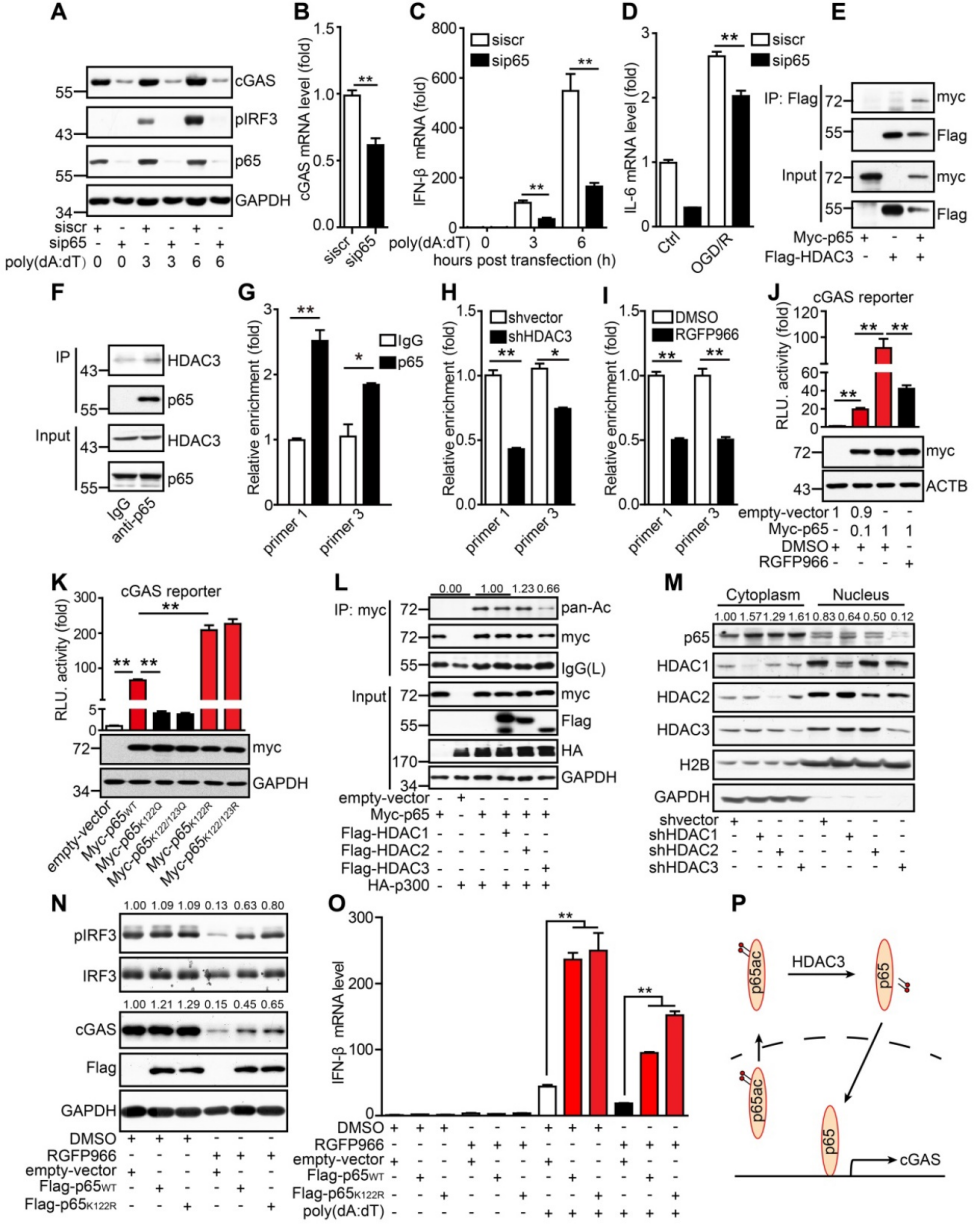
** HDAC3 promotes cGAS transcription by deacetylating p65.** (**A**) BV2 cells transfected with siRNA against p65 or scrambled siRNA were treated with poly(dA:dT)-Lipofectamine 2000 complex for 3 h and the expression of pIRF3, cGAS, p65, and GAPDH was analyzed. (**B**) The mRNA level of cGAS in p65-silenced and control cells was quantified by real-time PCR. (**C**) BV2 cells transfected with siRNA against p65 or scramble were treated with poly(dA:dT)-Lipofectamine 2000 complex for 6 h and the transcription levels of IFN-β and β-actin were analyzed by real-time PCR. (**D**) p65-silenced and control cells that underwent OGD/R were collected and the expression of IL-6 was analyzed by real-time PCR. (**E**) pFlag-HDAC3 and pMyc-p65 plasmids were co-transfected into HEK293T cells, and the cells were harvested for Co-IP with anti-Flag tag magnetic beads. (**F**) Co-IP was performed with a rabbit anti-p65 antibody or control rabbit IgG with BV2 cells. (**G**) The binding sites of p65 in the promoter region were analyzed by chromatin immunoprecipitation and real-time PCR with an antibody against p65. (**H** and **I**) The binding activity of p65 in the promoter region of cGAS in HDAC3-inhibited (H) and HDAC3-silenced (I) BV2 cells. (**J**) Cells were co-transfected with pMyc-p65, cGAS reporter plasmid and TK-Renilla plasmids, treated with RGFP966 or vehicle, and harvested 24 h post-transfection for the dual-luciferase assay. (**K**) Plasmids encoding WT p65, mutant p65_K122Q_, mutant p65_K122/123Q_, mutant p65_K122R_, or mutant p65_K122/123R_ were transfected into HEK293T cells with the cGAS reporter and TK-Renilla reference plasmids, and the cells were harvested for the dual-luciferase assay 24 h post-transfection. (**L**) Plasmids encoding Myc-p65 and HA-p300 and empty vectors were co-transfected into HEK293T cells with plasmids encoding Flag-HDAC1, Flag-HDAC2, and Flag-HDAC3 and empty vectors, and the acetylation level of p65 was analyzed with anti-acetylated lysine (pan-Ac) after p65 was purified with anti-Myc tag magnetic beads. (**M**) The cytoplasm and nucleus of HDAC1-silenced, HDAC2-silenced, HDAC3-silenced, and control BV2 cells were separated and the expression of p65 was analyzed by western blot. (**N** and **O**) BV2 cell lines stably expressing Flag-tagged WT p65 or mutant p65_K122R_ or control cell lines were pretreated with RGFP966 for 12 h, transfected with poly(dA:dT), and harvested to analyze the protein levels of pIRF3, cGAS, p65, and GAPDH (N) and the mRNA level of IFN-β (O). (**P**) Schematic showing how HDAC3 regulates the transcription of cGAS by deacetylating p65. (* indicates* p* < 0.05, ** indicates* p* < 0.01 by ANOVA or Student's *t*-test). Abbreviations: cGAS, cyclic GMP-AMP synthase; Co-IP, co-immunoprecipitation; GADPH, glyceraldehyde 3-phsophate dehydrogenase; HDAC3, histone deacetylase 3; IFN-β, interferon-beta; OGD/R, oxygen-glucose deprivation/reperfusion; pIRF3, phosphorylated interferon regulatory factor 3; siRNA, small interfering RNA; WT, wild-type.

These results indicate that HDAC3 positively regulates the transcription of cGAS in microglia. To further decipher the mechanism underlying this, we knocked down several candidate downstream targets of HDAC3 such as forkhead box protein O3A, transcription factor Sp1, and p65, all of which have been reported to be actively involved in oxidative stress responses. In our experiments, only the knockdown of p65 in microglia decreased protein and mRNA levels of cGAS (Figure 5A, B). The phosphorylation levels of IRF3 (Figure 5A) and transcriptional levels of IFN-β (Figure 5C) were both reduced in p65-silenced BV2 cells. Accordingly, the OGD/R-induced activation of cGAS was also suppressed in p65-silenced BV2 cells (Figure 5D and Figure S6A). Using the exogenous and endogenous Co-IP assay, we found that HDAC3 interacted with p65 in HEK293T cells (Figure 5E), and microglia (Figure 5F). Further ChIP analysis showed that p65 bound to the cGAS promoter (Figure 5G), and this interaction was reduced in HDAC3-silenced cells (Figure 5H) and RGFP966-treated cells (Figure 5I) microglia. Furthermore, overexpression of p65 upregulated the activity of the cGAS promoter, and this upregulation was inhibited by HDAC3 inhibition or pan-HDAC inhibitors (Figure 5J and Figure S6B).

It has been reported that lysine acetylation at sites 122 and 123 of p65 results in the cytoplasmic translocation of p65 from the nucleus. This is followed by the IκB-mediated degradation of p65, which inhibits its DNA-binding activity. Here, we determined whether the acetylation of p65 at K122 and K123 inhibited the binding of p65 to the cGAS promoter. When lysine was substituted with glutamine at site 122 (K122Q), which mimics the acetylated form, the transcriptional activity of p65_K122Q_ on the cGAS promoter was almost abolished. In contrast, when lysine was substituted with arginine (K122R), which mimics the unacetylated form, the transcriptional activity of p65_K122R_ on the cGAS promoter was significantly increased (Figure 5K). Moreover, we found that acetylation of p65 at K310 had no effect on the transcription of cGAS (Figure S6C). The acetylation level of p65_K122R_ was much lower than that of WT p65 (Figure S6D). Interestingly, overexpression of HDAC3 attenuated the p300-mediated acetylation of p65 (Figure 5L). Further subcellular fractionation analysis revealed that the nuclear accumulation of p65 markedly decreased when HDAC3 was knocked down (Figure 5M), and its activity was inhibited by RGFP966 (Figure S6E). In contrast, the nuclear localization of p65 was promoted by p65_K122R_ and inhibited by p65_K122Q_ (Figure S6F). These results suggest that acetylation of p65 at K122 impairs its nuclear translocation and DNA-binding activity and that HDAC3 can increase p65 transactivation by deacetylating p65 at K122.

We then generated stable BV2 cell lines overexpressing WT p65 (p65_WT_) or K122R mutant p65 (p65_K122R_). In BV2 cells, RGFP966 treatment reduced cGAS expression and IRF3 phosphorylation, and these reductions were significantly attenuated by the overexpression of p65 (Figure 5N). Notably, the attenuating effect of p65_K122R_ was marginally higher than that of p65_WT_, especially with respect to IRF3 phosphorylation (Figure 5N). Similarly, under poly(dA:dT) stimulation, p65_WT_ and p65_K122R_ overexpression dramatically upregulated the expression of IFN-β, and this was significantly reduced by HDAC3 inhibition (Figure 5O). Compared to p65_WT_, p65_K122R_ overexpression attenuated the RGFP966-induced inhibition of IFN-β expression to a greater degree (Figure 5O). Taken together, we argue that acetylation of p65 at K122 inhibits its nuclear localization and transactivation, thus inhibiting cGAS transcription and dampening cGAS signaling in microglia. Importantly, HDAC3 upregulates cGAS transcription by deacetylating p65 at K122 and increasing its nuclear accumulation (Figure 5P).

### Microglial HDAC3 deficiency suppresses cGAS-STING-mediated autoimmune response *in vivo*

**Figure 6:**
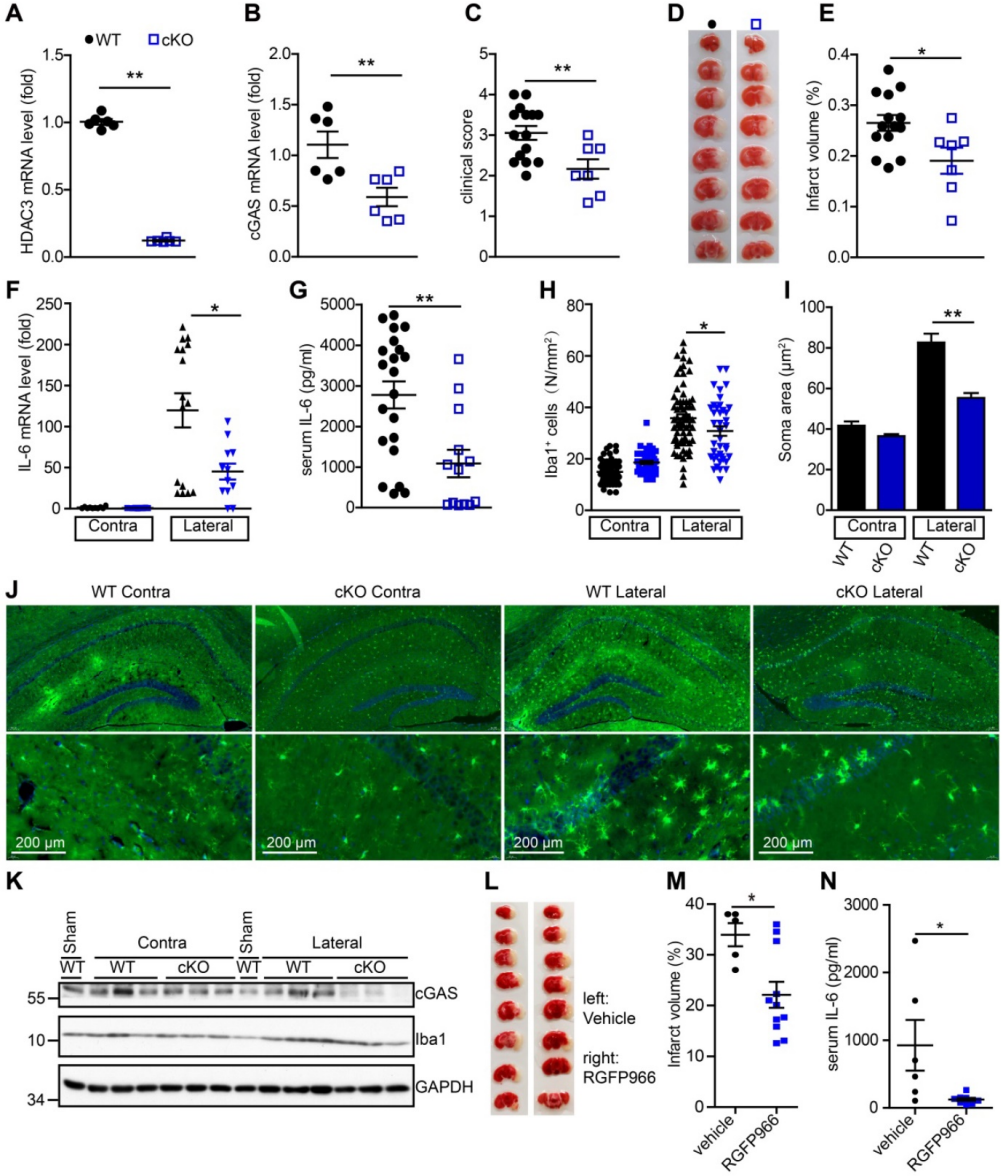
** Deficiency of HDAC3 in microglia suppresses the cGAS-STING-mediated autoimmune response**. (**A** and **B**) Adult primary microglial cells were isolated from HDAC3 cKO (n = 6) and WT (n = 6) mice, and the mRNA levels of HDAC3 (A) and cGAS (B) were analyzed by real-time PCR. (**C**) The neurological deficits of HDAC3 cKO (n = 7) and WT (n = 15) mice subjected to tMCAO were assessed 24 h post-reperfusion. (**D** and **E**) Brain tissue isolated from WT (n = 14) and HDAC3 cKO (n = 7) mice 24 h post-reperfusion was stained with triphenyl tetrazolium chloride, and infarct volume was determined using ImageJ (D) and infarct volume was determined using Image J (E). (**F**) RNA was extracted from the ischemic penumbra cortex and contra cortex of HDAC3 cKO (n = 12) and WT (n = 14) mice subjected to tMCAO 6 h post-reperfusion, and the mRNA level of IL-6 was quantified by real-time PCR. (**G**) Serum was isolated from HDAC3 cKO (n = 13) and WT (n = 21) mice subjected to tMCAO 6 h post-reperfusion, and the level of IL-6 was quantified by ELISA. (**H** to **J**) The number (**H** and **J**) and soma area (**I** and **J**) of Iba1^+^ cells in the ischemic penumbra and contralateral cortex of HDAC3 cKO and WT mice subjected to tMCAO 24 h post-reperfusion. (**K**) Proteins extracted from the ischemic penumbra and contralateral cortex of HDAC3 cKO and WT mice subjected to tMCAO 6 h post-reperfusion were analyzed with antibodies against cGAS, HDAC3, Iba1, and GAPDH. (**L** and **M**) Mice administered RGFP966 (n = 11) or vehicle (n = 5) for 2 days underwent tMCAO, and infarct volume was determined by TTC staining. (**N**) Serum was isolated from RGFP966-treated (n = 8) and vehicle-treated (n = 6) mice subjected to tMCAO 6 h post-reperfusion and the level of IL-6 was quantified by ELISA. (* indicates* p* < 0.05, ** indicates* p* < 0.01 by ANOVA or Student's *t*-test). Abbreviations: cGAS, cyclic GMP-AMP synthase; cKO, conditional knockout; ELISA, enzyme-linked immunosorbent assay; GAPDH, glyceraldehyde 3-phosphate dehydrogenase; HDAC3, histone deacetylase 3; Iba1, ionized calcium-binding adapter molecule 1; IL-6, interleukin-6; STING, stimulator of interferon genes; tMCAO, transient middle cerebral artery occlusion; TTC, triphenyl tetrazolium chloride; WT, wild-type.

To further study the role of microglial HDAC3 in I/R-induced activation of the cGAS-STING pathway and brain injury, we generated HDAC3^f/f^:CX3CR1^creER/+^ mice by crossing HDAC3^f/f^ mice with CX3CR1^creER/+^ mice. In these mice, tamoxifen administration induced the cKO of HDAC3 in microglia. The mRNA level of HDAC3 was significantly decreased in adult primary microglia from HDAC3 cKO mice 2 weeks after tamoxifen administration (Figure 6A). Consistently, the number of ionized calcium-binding adapter molecule 1 (Iba1) and HDAC3 double-positive cells was reduced in the cortex of HDAC3 cKO mice (Figure S7A) and protein levels of HDAC3 in adult primary microglia also decreased (Figure S7B). These results suggested that HDAC3 was almost completely deleted in microglia from cKO mice.

Following this, we found that the expression of cGAS was significantly reduced in microglia from HDAC3 cKO mice 2 weeks after tamoxifen administration (Figure 6B). Since cGAS-STING signaling is actively involved in I/R-induced neuroinflammation and brain injury, we sought to study the role of microglial HDAC3 in I/R-induced brain injury and neuroinflammation. We found that HDAC3 cKO mice exhibited a lower neurological deficit score (Figure 6C) and a smaller infarct volume (Figure 6D, E), demonstrating that depleting HDAC3 in microglia protected against I/R-induced brain injury. Furthermore, mRNA levels of IL-6 in the ischemic penumbra of HDAC3 cKO mice were lower than that in the penumbra of WT mice (Figure 6F). Moreover, the serum levels of IL-6 post-I/R were also lower in HDAC3 cKO mice (Figure 6G). Interestingly, we found that microglial activation was also decreased in the ischemic penumbra of HDAC3 cKO mice, as demonstrated by the presence of fewer Iba1^+^ cells (Figure 6H), and the fact that microglia exhibited a smaller Soma area (Figure 6I, J and Figure S7C). We also observed decreased protein levels of cGAS in brain tissue from HDAC3 cKO mice (Figure 6K). Because RGFP966 is a blood-brain barrier-permeable chemical, we found that RGFP966 administration overtly reduced the expression of cGAS in the cortex (Figure S7D). Importantly, administration of RGFP966 significantly decreased the infarct volume of WT mice subjected to tMCAO (Figure 6L, M). Similar to the knockout of HDAC3 in microglia, inhibition of HDAC3 with RGFP966 reduced mRNA levels of IL-6 in the ischemic penumbra in WT mice (Figure S7E) and serum levels of IL-6 (Figure 6N). Taken together, these data suggest that *in vivo* HDAC3 inhibition can downregulate the expression and activity of cGAS and is beneficial for the treatment of I/R-induced tissue injury.

**Figure 7:**
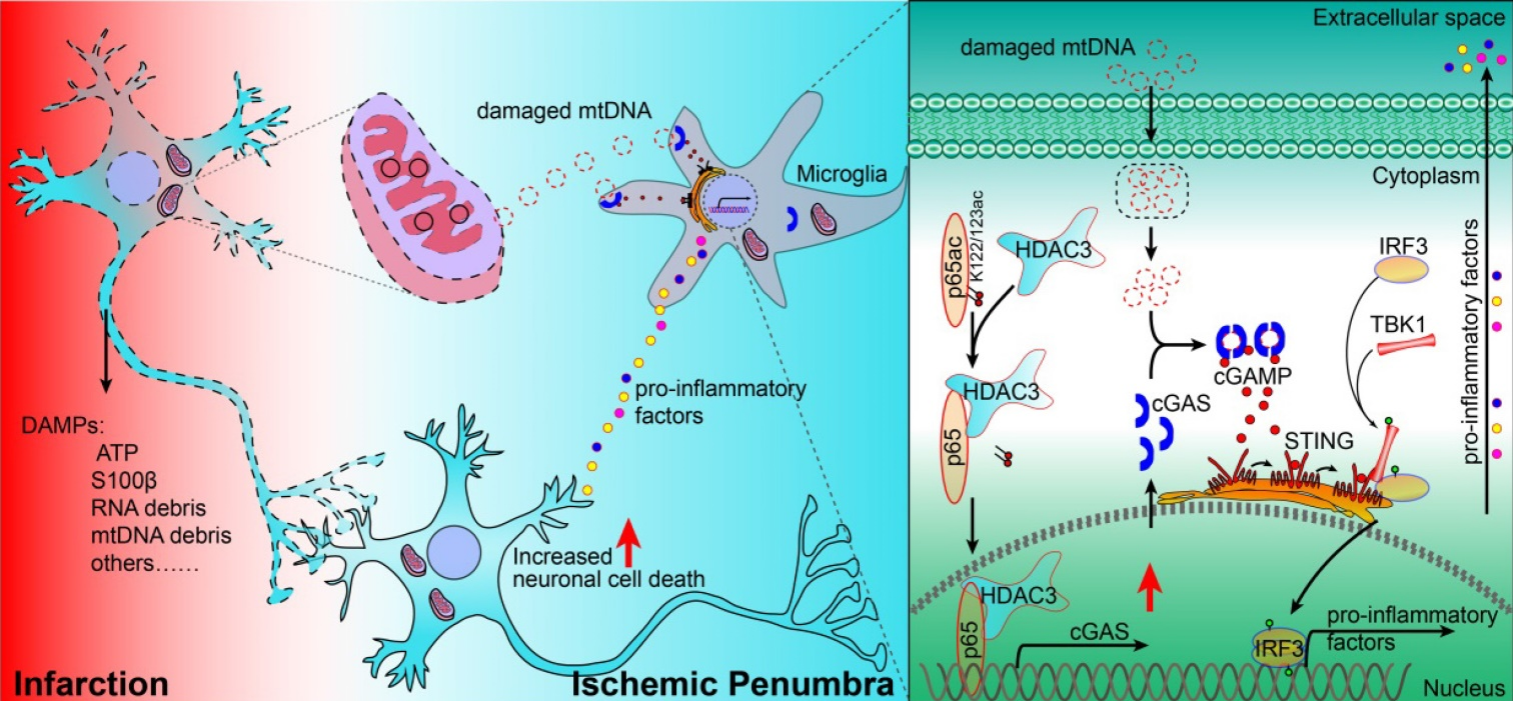
** Proposed mechanism: HDAC3 regulates the transcription of cGAS by deacetylating p65 at K122, which plays a role in ischemic/reperfusion-induced neuroinflammation and brain injury.** Briefly, mtDNA released from dead cells in the ischemic core is engulfed by microglia in the penumbra. Toxic mtDNA then activates the microglial cGAS-STING-IRF3 pathway and potentiates neuroinflammation, which in turn aggravates ischemia-induced neuronal cell death. Within microglia, HDAC deacetylates p65, which promotes its nuclear translocation and induces cGAS expression and cGAS-STING pathway activation. Abbreviations: cGAS, cyclic GMP-AMP synthase; HDAC, histone deacetylase; IRF3, interferon regulatory factor 3; mtDNA, mitochondrial DNA; STING, simulator of interferon genes.

In summary, our study revealed that the cGAS-STING pathway is involved in I/R-induced neuroinflammation and brain injury. Microglial deletion of cGAS can attenuate neuroinflammation and brain injury post-I/R. Mechanistically, HDAC3 deacetylates p65 and transcriptionally upregulates the expression of cGAS, which is needed to promote cGAS-STING signaling activation and neuroinflammation. Microglial deletion of HDAC3 or HDAC3 inhibition attenuates cGAS-STING pathway activation and prevents neuroinflammation and brain injury induced by cerebral I/R (Figure 7).

## Discussion

DNA and RNA sensors have been shown to mediate some autoimmune disorders and activate germ-free inflammation in some diseases 4, 17, 36. In this study, we discovered that the DNA-sensing and type I interferon pathways were activated and ISGs were upregulated in microglia after ischemic stroke (Figure 1A-C). IRF3 and NF-κB are activated upon cGAS activation by cytosolic DNA to mediate the transcription of inflammatory factors. Canonically, p65 forms heterodimers with p50 to activate the transcription of pro-inflammatory genes. Additionally, p65 could form homodimers and activate the transcription of *interleukin-18* independent of p50 37, 38. In the current study, we found that p65 might regulate the transcription of cGAS in a non-canonical manner. Knockdown of p65 resulted in decreased expression of cGAS, which impaired the activation of the downstream type I interferon pathway (Figure 5A-C). These results suggest that p65 may be an upstream regulator of cGAS transcription.

Moreover, we found that the expression of cGAS was increased in microglia during the early stage after I/R (Figure 2A, B). The *in vitro* and *in vivo* experiments indicated that cGAS was involved in the activation of the type I interferon pathway during the first few hours after I/R (Figure 2G-K). The DNA sensor cGAS is usually activated by pathogen-derived DNA but is also activated by self-mtDNA and gDNA in some autoimmune disorders or tumor cells. In our study, we found that cGAS was most likely activated by mtDNA released from dead cells or the mitochondria of cells subjected to I/R, OGD/R, or oxidative stress. The amount of cGAS-bound mtDNA in cells subjected to I/R, OGD/R, or oxidative stress was significantly increased (Figure 2H, I and Figure S2F). Recently, other research groups proposed that self-mtDNA can activate the cGAS-STING pathway, and suggested that the activation of cGAS is involved in high-fat diet-induced obesity and tumor growth 18, 39. In the current study, we found that cGAS directly binds to mtDNA in the ischemic penumbra of mice subjected to I/R injury and OGD/R-treated microglial cells, which promotes the activation of the type I interferon pathway. Evidence from IFNAR1, IFNAR2, and IRF3 knockout mice subjected to tMCAO indicated that activation of the type I interferon pathway plays a detrimental role after stroke by creating a pro-inflammatory environment 8, 9. Here, downregulation of cGAS in microglia mitigated OGD/R- and I/R-induced neuroinflammation and attenuated I/R-induced brain injury, which strongly suggests that cGAS could be a potential drug target for the treatment of stroke.

Previous studies have indicated that post-translational modifications, such as phosphorylation, glutamylation and ubiquitination, play critical roles in regulating the activity and stability of cGAS 23, 24, 40, 41. However, these regulators are not feasible targets for drug development because they have multiple targets. In the present study, inhibition of HDAC3 blocked the cGAS-mediated activation of the type I interferon pathway. Interestingly, HDAC3 specifically promoted the transcription of cGAS in a p65-dependent manner, which is consistent with the findings of Ziesche et al. 42. Importantly, our data clearly showed that microglial HDAC3-deficiency impaired the nuclear accumulation and cGAS promoter-binding activity of p65 (Figure 5H, I, L, and M). In contrast, acetylation of p65 at site 122 prevented its nuclear translocation and cGAS promoter-binding activity (Figure 5J, K and Figure S6F). Thus, our findings elucidated a previously unidentified regulatory mechanism of cGAS expression in microglia. Interestingly, HDAC3 also regulated cGAMP-induced activation of the STING-TBK1-IRF3 cascade (Figure S5). Since there is a lack of effective inhibitors of cGAS and STING, our data suggest that HDAC3 inhibitors could be novel drugs for the treatment of ischemic brain injury due to their downregulation of cGAS-STING-mediated neuroinflammation.

Increasing evidence has shown that inhibition of HDACs promotes endogenous neuronal regeneration and protects neuronal cells from I/R-induced apoptosis 29, 30, 32. Our previous study showed that oxidative stress-induced phosphorylation of HDAC2 is involved in oxidative stress-induced neuronal cell death. Recently, it has been shown that the expression of HDAC3 is upregulated in brain tissue after I/R 43. There is a decreased expression level of pro-inflammatory factors in HDAC3 knockout macrophages, which is similar to IL-4 treatment 44, 45. These studies suggest that HDAC3 is a positive regulator of inflammation. In the current study, we focused on the role of HDAC3 in microglia-mediated neuroinflammation during ischemic stroke and found that HDAC3 deficiency in microglia ameliorated acute I/R-induced microglial activation and inflammation, indicating that targeting HDAC3 may be a novel strategy for the treatment of ischemic stroke.

## Conclusions

In summary, our study revealed that the microglial cGAS-STING pathway is activated by mtDNA, which promotes the formation of a pro-inflammatory microenvironment. HDAC3 transcriptionally upregulates the expression of cGAS and potentiates the activation of the cGAS-STING pathway in microglia. The expression of cGAS and the activity of the cGAS-STING pathway are regulated by HDAC3 in a p65-dependent manner. Deficiency of HDAC3 in microglia decreases the nuclear localization of p65, which downregulates the expression of cGAS and the activation of cGAS-STING pathway. Overexpression of p65 partially rescued the expression and activity of cGAS when the activity of HDAC3 is inhibited. Notably, our study proved that p65 positively regulates the activation of the type I interferon pathway through cGAS. Furthermore, the *in vivo* results indicated that deletion of cGAS or HDAC3 in microglia can attenuate I/R-induced brain injury and neuroinflammation. Therefore, the HDAC3-p65-cGAS-STING pathway may be a therapeutic target for ischemic stroke.

## Supplementary Material

Supplementary figures and tables.

## Abbreviations

cGAMP: cyclic GMP-AMP
cGAS: cyclic GMP-AMP synthase
STING: stimulator of interferon genes
HDAC: histone deacetylase
IFN-β: interferon-beta
IL-6: interleukin 6
IRF3: interferon regulatory factor 3
TBK1: Tank-binding kinase 1
IFNAR: Interferon alpha/beta receptor
NFκB: nuclear factor kappa-B
GAPDH: Glyceraldehyde-3-phosphate dehydrogenase
ACTB: beta-actin
mtDNA: mitochondrial DNA
gDNA: genomic DNA
tMCAO: transient middle cerebral artery occlusion
I/R: ischemia/reperfusion
OGD/R: oxygen-glucose deprivation/reperfusion
cKO: conditional knockout
FBS: fetal bovine serum
PBS: phosphate-buffered saline
Co-IP: co-immunoprecipitation
