# Supplementary material for: HDAC3 inhibition ameliorates ischemia/reperfusion-induced brain injury by regulating the microglial cGAS-STING pathway
Source: Theranostics. 2020 Jul 29;10(21):9644–62. doi: 10.7150/thno.47651 (PMC7449914; doi:10.7150/thno.47651)
Supplement: Supplementary file 1 — Supplementary figures and tables. [file thnov10p9644s1.pdf]

Supplementary Materials:

Tables

Table S1. Sequences of the primers used in this study.

Primer	Sequence (5′-3′)	Usage
cGAS (up2000 reporter)	Sense: CCG*CTCGAG*CTGGAGCTACGGACCTATGC<br>Antisense: CCC*AAGCTT*CGAAGAAAGGCCGCGAAAAG	Cloning the 2000 bp upstream of the cGAS initial codon into the pGL3-basic vector
HDAC3 (mouse)	Sense: GC*TCTAGA*ATGGCCAAGACCGTGGCCTATTTC<br>Antisense: CG*GGATCC*CTAAATCTCCACATCGCTTTCC	Cloning HDAC3 into the pFlag-CMV-10 vector
$p65_{K122Q}$	Sense: GTGCAGAAGCGGGACCTGGAGC<br>Antisense: GGTCCCGCTTCTGCACACACTGGAT	Constructing the p65 K122Q mutant plasmid
$p65_{K122R}$	Sense: GTGAGGAAGCGGGACCTGGAGC<br>Antisense: GGTCCCGCTTCCTCACACACTGGAT	Constructing the p65 K122R mutant plasmid
$p65_{K122/123Q}$	Sense: GTGCAGCAGCGGGACCTGGAGC<br>Antisense: GGTCCCGCTGCTGCACACACTGGAT	Constructing the p65 K122/123Q mutant plasmid
$p65_{K122/123R}$	Sense: GTGAGGAGGCGGGACCTGGAGC<br>Antisense: GGTCCCGCCTCCTCACACACTGGAT	Constructing the p65 K122/123R mutant plasmid
$p65_{K310Q}$	Sense: GACCTTCCAGAGCATCATGAAGAAG<br>Antisense: CTCTGGAAGGTCTCATATGTCCTTTTACG	Constructing the p65 K310Q mutant plasmid
$p65_{K310/314/315Q}$	Sense:<br>CCTTCCAGAGCATCATGCAGCAGAGTCCTTTCA<br>Antisense:<br>CTGCTGCATGATGCTCTGGAAGGTCTCATATGTCC	Constructing the p65 K310/314/315Q mutant plasmid
$p65_{K310R}$	Sense: GACCTTCAGGAGCATCATGAAGAAG<br>Antisense: CTCCTGAAGGTCTCATATGTCCTTTTACG	Constructing the p65 K310R mutant plasmid
GAPDH-qPCR (mouse)	Sense: AGGTCGGTGTGAACGGATTTG<br>Antisense: GGGGTCGTTGATGGCAACA	Real-time PCR
cGAS-qPCR	Sense: GTCGGAGTTCAAAGGTGTGGA<br>Antisense: GACTCAGCGGATTTCCTCGTG	Real-time PCR
IFN-β-qPCR (mouse)	Sense: GCACTGGGTGGAATGAGACTATTG<br>Antisense: TTCTGAGGCATCAACTGACAGGTC	Real-time PCR

IL-6-qPCR (mouse)	Sense: GCTACCAAACTGGATATAATCAGGA<br>Antisense: CCAGGTAGCTATGGTACTCCAGAA	Real-time PCR
iNOS-qPCR (mouse)	Sense: GTTCTCAGCCCAACAATACAAGA<br>Antisense: GTGGACGGGTCGATGTCAC	Real-time PCR
ISG20-qPCR (mouse)	Sense: TGGGCCTCAAAGGGTGAGT<br>Antisense: CGGGTCGGATGTACTTGTCATA	Real-time PCR
ACTB-qPCR (mouse)	Sense: GGCTGTATTCCCCTCCATCG<br>Antisense: CCAGTTGGTAACAATGCCATGT	Real-time PCR
HDAC3-qPCR (mouse)	Sense: AGTTCTGCTCCCGTTACACA<br>Antisense: TAAGCAGCTCCAGGATACCAATT	Real-time PCR
mtDNA 1 (mouse_COXI)	Sense: GCCCCAGATATAGCATTCCC<br>Antisense: GTTCATCCTGTTCCTGCTCC	Real-time PCR
gDNA1 (mouse_TerT)	Sense: CTAGCTCATGTGTCAAGACCCTCTT<br>Antisense: GCCAGCACGTTTCTCTCGTT	Real-time PCR
mtDNA 2 (mouse_UUR)	Sense: GGCAGAGCCAGGAAATTGC<br>Antisense: CACTATTAGGGAGAGGATTTGAACCT	Real-time PCR
gDNA2 (mouse_B2M)	Sense: ATCCTGGCTCACACTGAATTCA<br>Antisense: TGCTTAACTCTGCAGGCGTATG	Real-time PCR
cGAS chip-qPCR P1	Sense: GCAAAATGAGTTCCGCCAAG<br>Antisense: TTGGCTGCTGAGATTCCGTA	Real-time PCR
cGAS chip-qPCR P2	Sense: AAAGTAGGCAGCGTTTCCAT<br>Antisense: CCTATTGACCCTGCAACTCT	Real-time PCR
cGAS chip-qPCR P3	Sense: TTGAAGACCAGGCATTAATC<br>Antisense: GACTTCCCAAACAGAAACTC	Real-time PCR
cGAS chip-qPCR P4	Sense: ACCGGACAAGCTAAAGAAGGTGCT<br>Antisense: GCAGCAGGCGTTCCACAACTTTAT	Real-time PCR (negative control)
GAPDH-qPCR	Sense: AAACCTGCCAAGTACGATGACA<br>Antisense: CCAGCCCCAGCGTCGAAG	Real-time PCR

(Macaca)		
cGAS-qPCR (Macaca)	Sense: TAAAGCCGTTTTACCTTGTACCCA<br>Antisense: ATTTCTTTGTTTTCACAGCACGTT	Real-time PCR
IFN-β-qPCR (Macaca)	Sense: AGCAGCAGTTTTCAGTGTCA<br>Antisense: TCTCATTCCAGCCAGTGCTA	Real-time PCR
cGAS-g	Sense: ATAGACCAAGCTGCTGCCCT<br>Antisense: GTTCCCAGCACCCTATCAGG	Genotyping
HDAC3-g	Sense: TGGTGGTGAATGGCTTTAATC<br>Antisense: TAACGGGAGCAGAACTCGAA	Genotyping
CX3CR1-creER	Common: CCGCCAGACGCCCAGACTA<br>Wildtype: AGCCGGAAGCCCAAGAGCATC<br>CreER-Tg: TGCTGCTGCCCGACAACCAC	Genotyping

Abbreviations: ACTB, ; B2M, ; cGAS, cyclic GMP-AMP synthase; COX1, ; CX3CR, ; GAPDH, glyceraldehyde 3-phosphate dehydrogenase; gDNA, genomic DNA; HDAC, histone deacetylase; IFN-β, interferon-beta; IL-6, interleukin-6; iNOS, inducible nitric oxide synthase; ISG20, ; mtDNA, mitochondrial DNA; qPCR, quantitative PCR; TerT, ; UUR, .

Table S2. Sequences of the siRNAs used in this study.

siRNA	Sequence (5′-3′)
sicGAS-a	GGAUUGAGCUACAAGAAUATT
sicGAS-b	GCUGUAACACUUCUUAUCATT
sip65-1	GAGUUUCAGCAGCUCCUGAACTT
sip65-2	GAAGCACAGATACCACCAATT
siSTING	CGAAAUAACUGCCGCCUCATT
siScr	UUUCCGAACGUGUCACGUTT

Abbreviations: cGAS, cyclic GMP-AMP synthase; Scr, scramble; siRNA, small interfering RNA; STING, stimulator of interferon genes.

Table S3. Sequences of the shRNAs used in this study.

shRNA	Sequence (5′-3′)
shvector	Sense:<br>CCGG*AGACACACGCACTCGTCTC*CTCGAG*AGACACACGCACTCGTCTC*TTTTTG<br>Antisense:<br>AATTCAAAAA*AGACACACGCACTCGTCTC*CTCGAG*AGACACACGCACTCGTCTC*
shHDAC1	Sense:<br>CCGG*GCAGATGCAGAGATTCAATGT*CTCGAG*GCAGATGCAGAGATTCAATGT*TTTTTG<br>Antisense:<br>AATTCAAAAA*GCAGATGCAGAGATTCAATGT*CTCGAG*GCAGATGCAGAGATTCAATGT*
shHDAC2	Sense:<br>CCGG*CGAGCATCAGACAAACGGATA*CTCGAG*CGAGCATCAGACAAACGGATA*TTTTTG<br>Antisense:<br>AATTCAAAAA*CGAGCATCAGACAAACGGATA*CTCGAG*CGAGCATCAGACAAACGGATA*
shHDAC3	Sense:<br>CCGG*CGTGGCTCTCTGAAACCTTAA*CTCGAG*CGTGGCTCTCTGAAACCTTAA*TTTTTG<br>Antisense:<br>AATTCAAAAA*CGTGGCTCTCTGAAACCTTAA*CTCGAG*CGTGGCTCTCTGAAACCTTAA*

Abbreviations: HDAC, histone deacetylase; shRNA, short hairpin RNA.

Supplementary figures and Figure legends

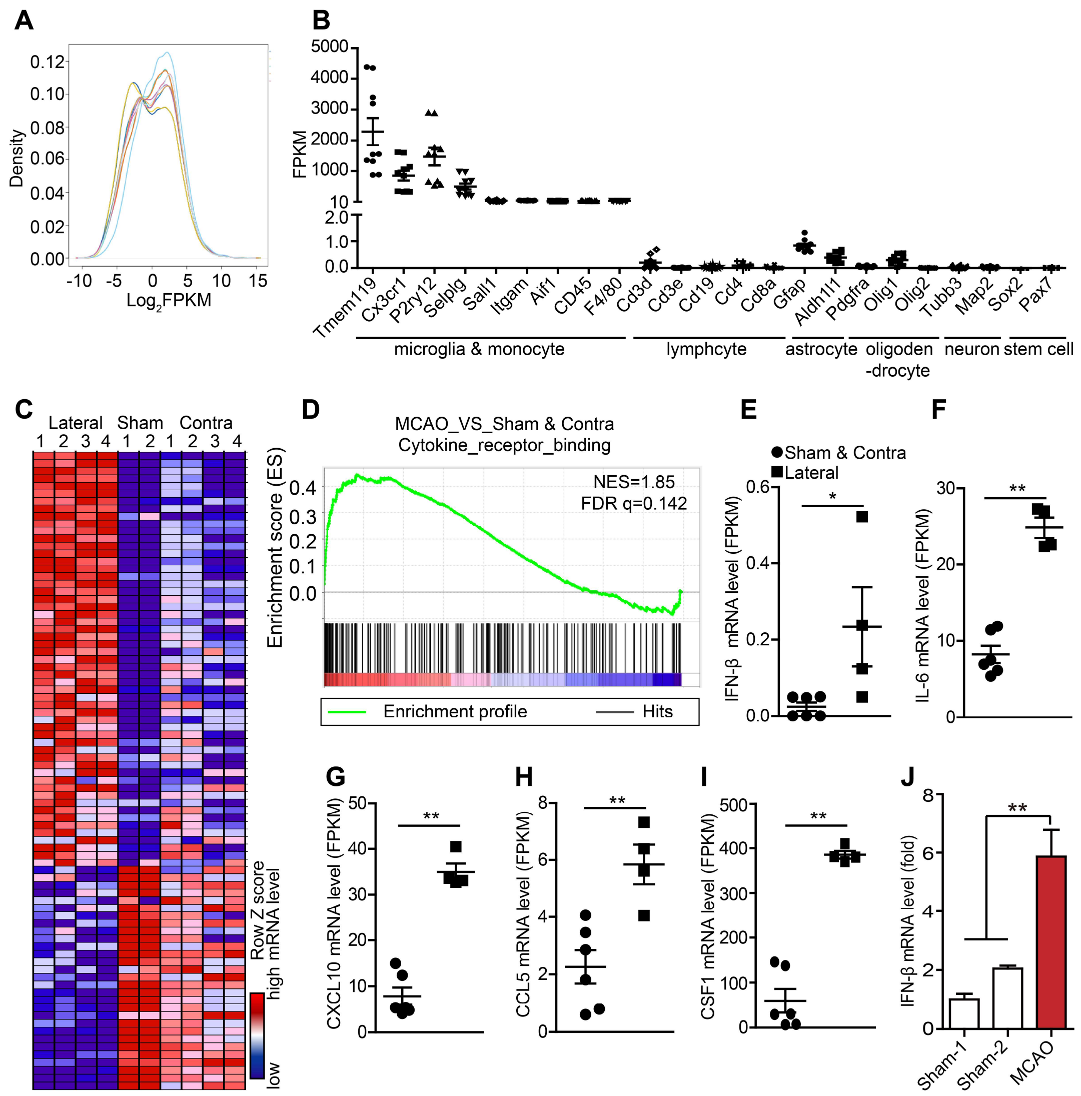

Supplementary Figure S1. **Associated with Fig. 1. The cGAS-STING pathway is involved in ischemic/reperfusion-induced neuroinflammation.** (A) Distribution of gene expression of RNA sequencing results in adult microglia isolated from brain. (B) The mRNA level of microglia-specific and other cell type specific genes in the isolated adult microglia for RNA sequencing. (C) Heatmap of up-/downregulated cytokines and cytokine receptors in primary microglia isolated from brain tissue extracted from mice subjected to tMCAO. (**D**) The cytokines and cytokine receptors were enriched from RNA-sequencing data by gene set enrichment analysis. (**E** to **I**) The mRNA levels of *ifn-β* (E), *il-6* (F), *cxcl10* (G), *ccl5* (H), and *csf1* (I) determined by RNA sequencing. (**J**) The mRNA level of IFN-β in the ischemic penumbra of Rhesus monkeys subjected to tMCAO or sham was analyzed by real-time PCR. (* indicates $p < 0.05$, ** indicates $p < 0.01$ by ANOVA or Student's *t*-test).

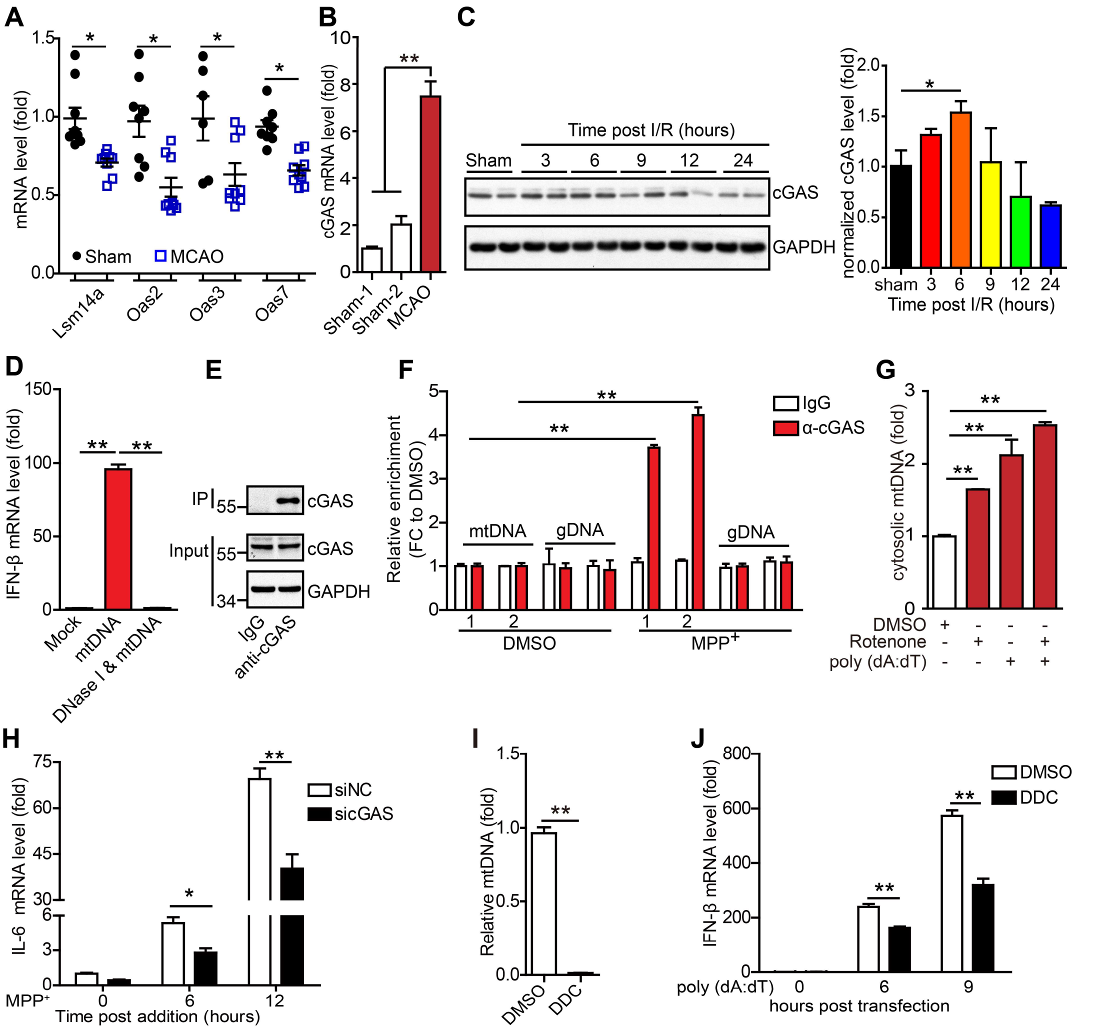

Supplementary Figure S2. Associated with Fig. 2. The cGAS-STING pathway is involved in oxidative stress-induced microglial activation. (**A**) The mRNA levels of some cytosolic DNA sensors in primary microglia isolated from mice that underwent tMCAO were quantified by real-time PCR. (**B**) The mRNA levels of cGAS in the ischemic penumbra cortex from Rhesus monkeys subjected to tMCAO or sham surgery were determined by real-time PCR. (**C**) The protein levels of cGAS in the ischemic penumbra cortex of mice that underwent tMCAO or sham surgery at 3, 6, 9, 12, and 24 h post-reperfusion were determined, and the gray values were analyzed by using ImageJ and were normalized to GAPDH. (**D**) BV2 cells transfected with mtDNA or DNase-pretreated mtDNA were collected to analyze the mRNA level of IFN-β. (**E**) cGAS-DNA complex was immunoprecipitated with a rabbit anti-cGAS polyclonal antibody, and normal rabbit IgG was used as a control. The protein levels of cGAS immunoprecipitated by the antibody or in the cell lysate were detected by western blot. (**F**) Control and MPP^{+}-treated BV2 cells were cross-linked and collected for analysis of the cGAS-bound mtDNA using a rabbit anti-cGAS polyclonal antibody and normal rabbit IgG. (**G**) mtDNA in the cytoplasm of cells treated with rotenone or poly(dA:dT) was isolated and quantified by real-time PCR. (**H**) cGAS-silenced and control BV2 cells were treated with MPP^{+} for 12 h and harvested for analysis of the mRNA level of IL-6. (**I**) BV2 cells were cultured with normal medium plus 20 μM

diethyldithiocarbamate for 6 days, and the amount of mtDNA was analyzed by real-time PCR. (**J**) Control and mtDNA-depleted BV2 cells stimulated with poly(dA:dT) for 0, 6, and 9 h were collected to determine the mRNA level of IFN-β. (* indicates $p < 0.05$, ** indicates $p < 0.01$ by ANOVA or Student's *t*-test).

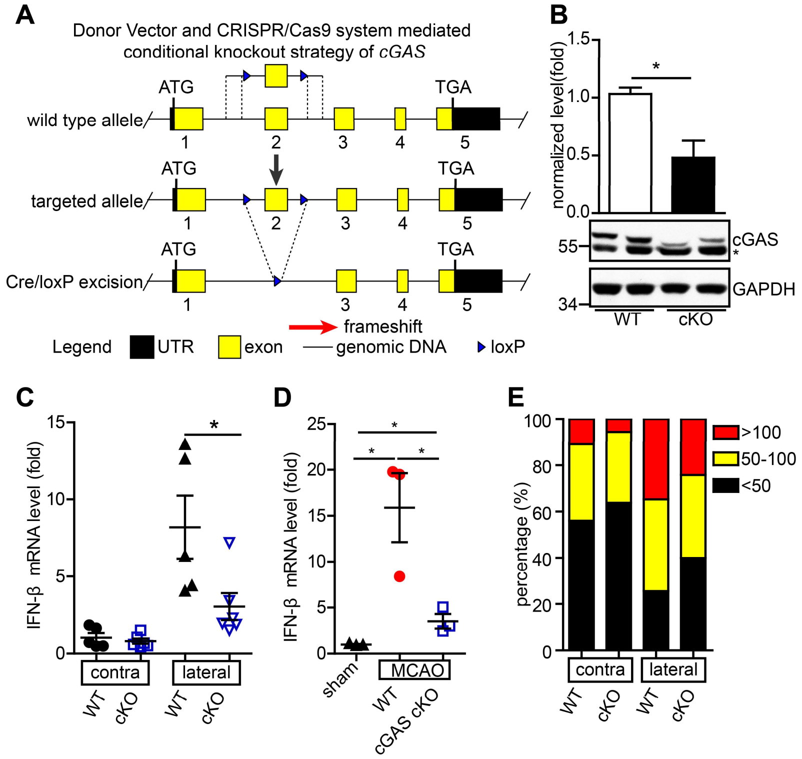

Supplementary Figure S3. Associated with Fig. 3. Microglial cGAS is involved in ischemic/reperfusion-induced neuroinflammation. (**A**) Schematic diagram of the strategy used to generate cGAS cKO mice. (**B**) Adult primary microglia were isolated from WT and cGAS cKO mice 2 weeks after tamoxifen administration, and the protein level of cGAS was quantified by western blot, and the gray values were analyzed by using ImageJ and were normalized to Iba1. (*: non-specific band). (**C**) The mRNA level of IFN-β in the ischemic and contralateral cortices of WT (n = 5) and cGAS cKO (n = 6) mice was analyzed by real-time PCR. (**D**) The mRNA level of IFN-β in microglia isolated from ischemic lateral of the brain of WT (n = 3) and cGAS cKO (n = 3) mice underwent sham or tMCAO operation was analyzed by real-time PCR. (**E**) The ratio of soma area greater than 100 μm^2 (red), between 50 and 100 μm^2 (yellow) and smaller than 50 μm^2 (black) of Iba1$^+$ cells in the ischemic and contralateral cortices of cGAS cKO and WT mice subjected to tMCAO 6 h post-reperfusion. (* indicates $p < 0.05$, ** indicates $p < 0.01$ by ANOVA or Student's *t*-test).

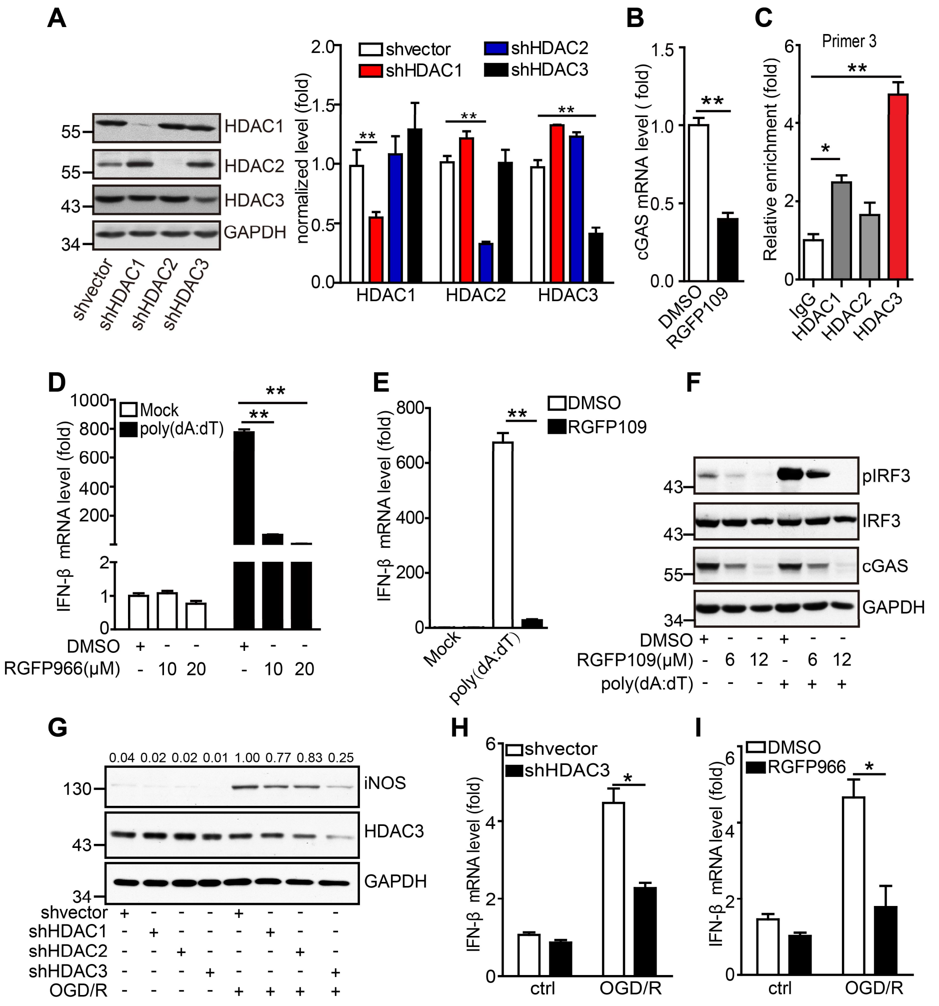

Supplementary Figure S4. Associated with Fig. 4. HDAC3 regulates the transcription of cGAS in microglia. (**A**) BV2 cells stably expressing shRNA against HDAC1, HDAC2, and HDAC3 were collected and the protein levels of the HDACs were analyzed by western blot, and the gray values were analyzed by using ImageJ and were normalized to GAPDH. (**B**) Control and RGFP109-treated BV2 cells were harvested for analysis of the mRNA level of cGAS by real-time PCR. (**C**) The enrichment of HDAC1, HDAC2, and HDAC3 in the promoter region of cGAS in BV2 cells was analyzed by chromatin immunoprecipitation followed by real-time PCR with antibodies against each HDAC. (**D** and **E**) RGFP966-pretreated (D), RGFP109-pretreated (E), and control BV2 cells transfected with poly(dA:dT) were harvested 6 h post-transfection for analysis of the mRNA level of IFN-β. (**F**) RGFP109-pretreated and control BV2 cells transfected with poly(dA:dT) were harvested 3h post-transfection for analysis of the protein levels of pIRF3, IRF3, cGAS, and GAPDH by western blot. (**G**) Control, HDAC1-silenced, HDAC2-silenced, and HDAC3-silenced BV2 cell lines that underwent OGD/R were harvested and the protein level of iNOS was analyzed by western blot, and the gray values were analyzed by using ImageJ and were normalized to GAPDH. (**H**) The mRNA level of IFN-β in control and HDAC3-silenced BV2 cells after OGD/R treatment. (**I**) The mRNA level of IFN-β in vehicle- and RGFP966-

pretreated cells after OGD/R treatment. (* indicates $p < 0.05$, ** indicates $p < 0.01$ by ANOVA or Student's *t*-test).

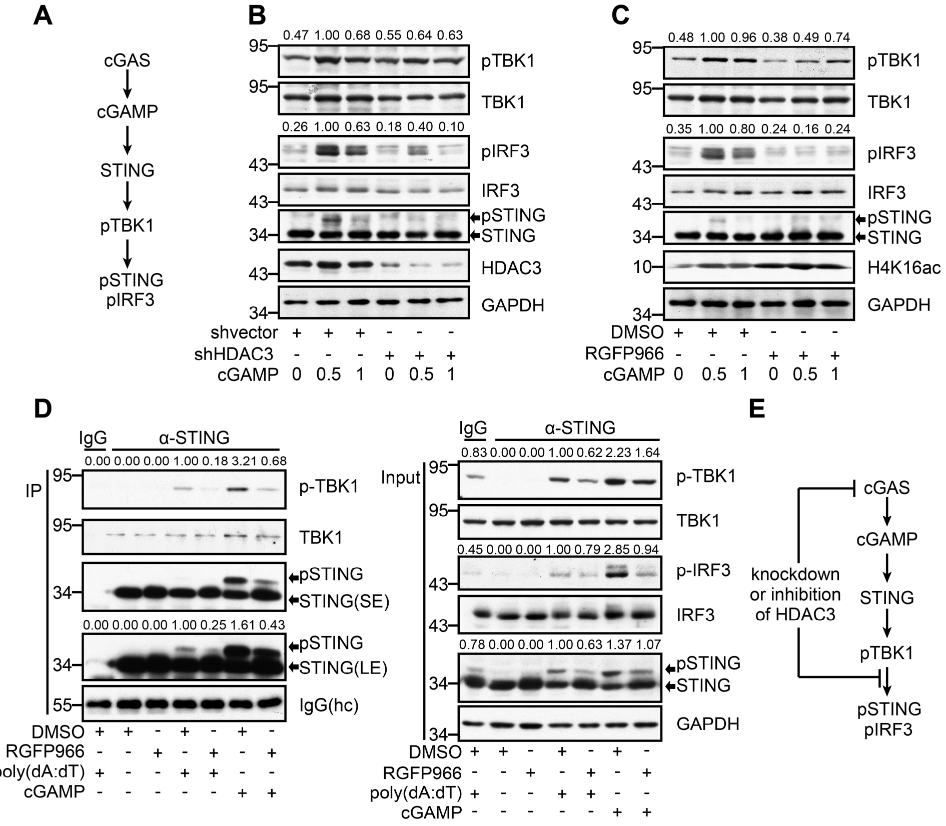

Supplementary Figure S5. HDAC3 functions as a downstream of cGAS and inhibition of microglial HDAC3 blocks the cGAMP-induced activation of the type I interferon pathway. (**A**) Schematic diagram of the cGAS-STING signaling pathway. (**B**) HDAC3-silenced and control BV2 cells were stimulated with cGAMP and the protein levels of pTBK1, pIRF3, TBK1, IRF3, STING, HDAC3, and GAPDH were analyzed by western blot, and the gray values were analyzed by using ImageJ and were normalized to GAPDH. (**C**) RGFP966-treated and control BV2 cells were stimulated with cGAMP and the protein levels of pTBK1, pIRF3, TBK1, IRF3, STING, acetylated H4 (H4K16ac), and GAPDH were analyzed by western blot, and the gray values were analyzed by using ImageJ and were normalized to GAPDH. (**D**) RGFP966-treated and control BV2 cells were stimulated with cGAMP or poly(dA:dT), were harvested for analysis the interaction between STING and TBK1 and the protein levels of pTBK1, pIRF3, TBK1, IRF3, STING, or GAPDH for IP complex and total cell lysates, and the gray values were analyzed by using ImageJ and were normalized to IgG heavy chain (IP) or GAPDH (input). (**E**) Schematic diagram of the cGAS-STING signaling pathway and its regulation by HDAC3.

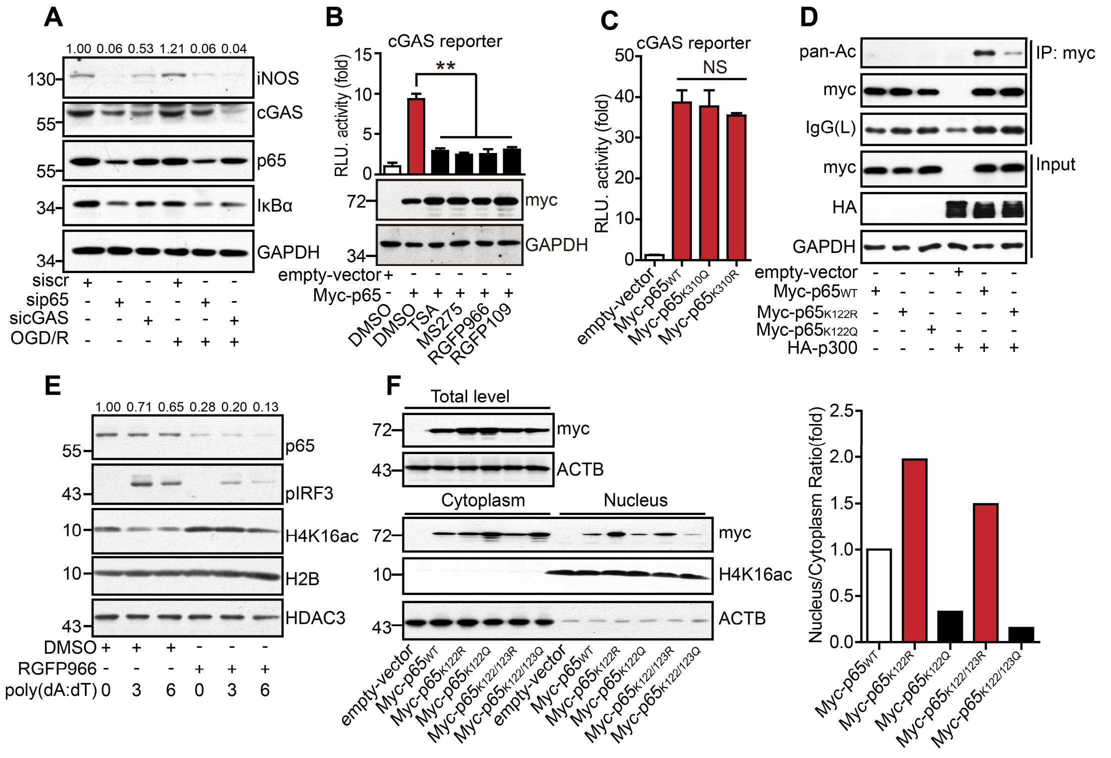

Supplementary Figure S6. Associated with Fig. 5. HDAC3 regulates the transcription of cGAS through the deacetylation of p65 in microglia. (**A**) p65-silenced, cGAS-silenced, and control cells that underwent OGD/R were collected and the expression of iNOS, cGAS, and p65 was analyzed by western blot, and the gray values were analyzed by using ImageJ and were normalized to GAPDH. (**B**) HEK293T cells transfected with Myc-p65, cGAS reporter, and TK-Renilla plasmids were treated with TSA, MS275, RGFP966, or RGFP109 for 24 h. Then, the cells were collected, the activity of the cGAS reporter was determined by a dual-luciferase assay, and the protein level of p65 was analyzed by western blot. (**C**) HEK293T cells transfected with plasmids encoding WT, K310Q, or K310R Myc-p65, cGAS, and TK-Renilla were collected and the activity of the cGAS reporter was determined by a dual-luciferase assay. (**D**) Empty vectors or plasmids encoding WT p65, mutant $p65_{K122Q}$, or mutant $p65_{K122R}$ were co-transfected into HEK293T cells with or without HA-p300, and the acetylation level of p65 was analyzed with anti-acetylated lysine (pan-Ac) after p65 was purified with anti-Myc tag magnetic beads. (**E**) The nucleus of RGFP966-treated and control BV2 cells were separated and the expression of p65 was analyzed by western blot, and the gray values were analyzed by using ImageJ and were normalized to H2B. (**F**) The cytoplasm and nucleus of HEK293T cells expressing Myc-tagged WT, K122R, K122Q, K122/123R, or K122/123Q p65 were separated, and the expression of p65 or mutant p65 was analyzed by western blot. (* indicates $p < 0.05$, ** indicates $p < 0.01$ by ANOVA or Student's *t*-test).

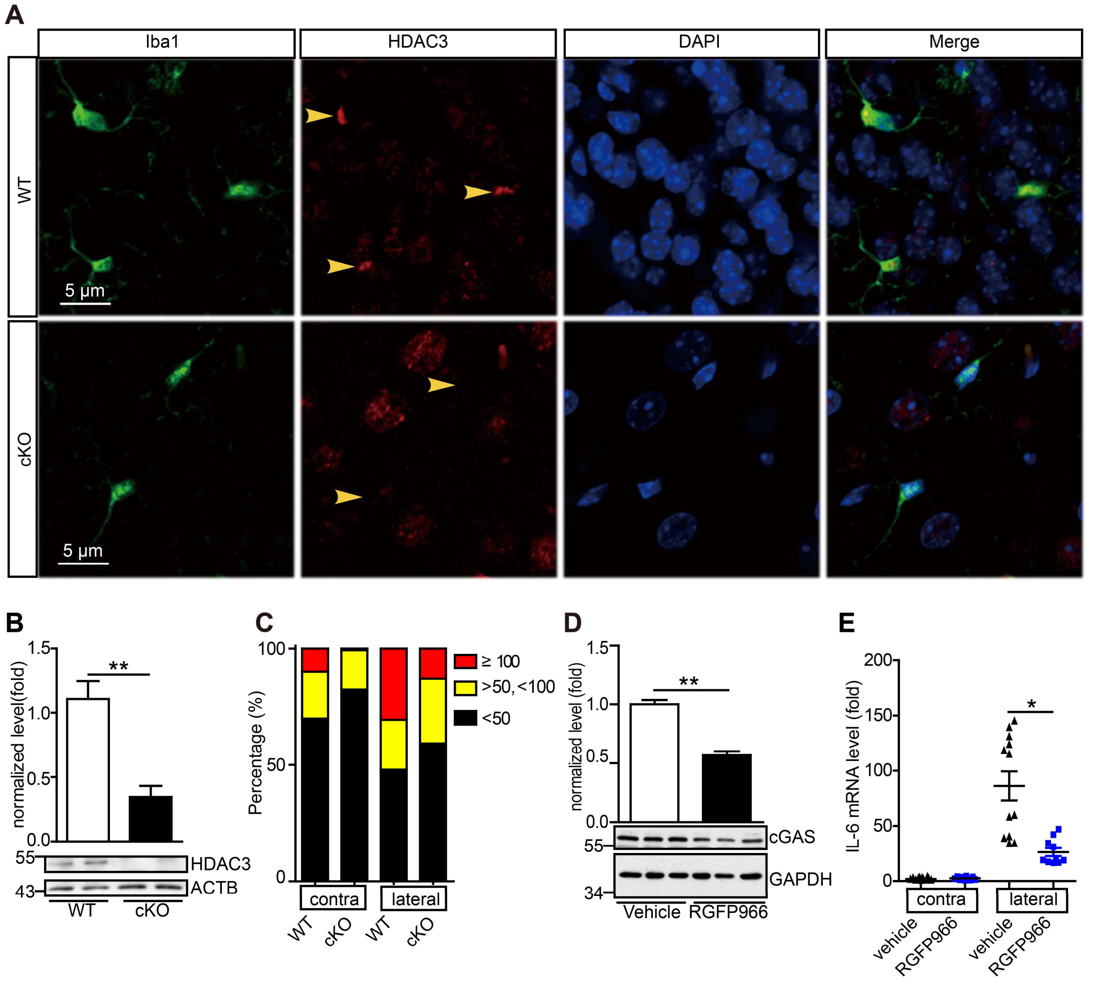

Supplementary Figure S7. Associated with Fig.6. HDAC3 is involved in ischemic/reperfusion-induced neuroinflammation. (**A**) Brain sections from WT and HDAC3 cKO mice were stained with a goat anti-Iba1 antibody (green) and rabbit anti-HDAC3 antibody (red), and images were captured using a confocal microscope. (**B**) Adult primary microglia were isolated from WT and HDAC3 cKO mice 2 weeks after tamoxifen administration, and the protein level of HDAC3 was quantified by western blot, and the gray values were analyzed by using ImageJ and were normalized to ACTB. (**C**) The ratio of soma area greater than 100 μm^2 (red), between 50 and 100 μm^2 (yellow) and smaller than 50 μm^2 (black) of Iba1$^+$ cells in the ischemic and contralateral cortices of HDAC3 cKO and WT mice subjected to tMCAO 6 h post-reperfusion. (**D**) Total protein was extracted from the cortex of mice treated with RGFP966 or vehicle for three consecutive days, and the protein level of cGAS was analyzed by western blot and normalized by ImageJ software. (**E**) RNA was extracted from the ischemic and contralateral cortices of RGFP966 (n = 10) and vehicle-treated (n = 12) mice subjected to tMCAO 6 h post-reperfusion, and the mRNA level of IL-6 was quantified by real-time PCR. (* indicates $p < 0.05$, ** indicates $p < 0.01$ by ANOVA or Student's *t*-test).
